# Differential modulation of gestational immunity by fatty acids: tissue-specific immune remodeling and clinical implications

**DOI:** 10.1042/CS20257900

**Published:** 2026-01-09

**Authors:** Jiajia Chen, Lingyu Chang, Xianyang Hu, Jiani Guo, Yang Yan, Dajin Li, Jinlong Qin, Meirong Du, Weijie Zhao

**Affiliations:** 1 Department of Gynecology and Obstetrics, Shanghai Fourth People's Hospital, School of Medicine, Tongji University, Shanghai, 200434, China; 2 Shanghai Key Laboratory of Maternal Fetal Medicine, Shanghai Institute of Maternal-Fetal Medicine and Gynecologic Oncology, Shanghai First Maternity and Infant Hospital, School of Medicine, Tongji University, Shanghai, 201204, China; 3 Laboratory of Reproduction Immunology, Obstetrics and Gynecology Hospital, Fudan University Shanghai Medical College, Shanghai, 200032, China; 4 Longgang District Maternity & Child Healthcare Hospital of Shenzhen City, Affiliated Shenzhen Women and Children’s Hospital (Longgang) of Shantou University Medical College, Shenzhen, 518172, China

**Keywords:** arachidonic acid, oleic acid, palmitic acid, maternal–fetal tolerance, miscarriage

## Abstract

Pregnancy necessitates dynamic maternal metabolic adaptations where fatty acids (FAs) serve dual roles as energy substrates and immunomodulators. However, the effects of specific FAs on gestational immunity and pregnancy outcomes remain elusive. In the present study, we administered saturated palmitic acid (PA), monounsaturated oleic acid (OA), polyunsaturated arachidonic acid (AA), or vehicle solutions daily to pregnant mice (gestational day 0.5 [GD0.5]–7.5) and performed comprehensive immune profiling at GD13.5. Mendelian randomization (MR) analysis was employed to evaluate translational relevance in human pregnancies. AA increased embryo resorption rates and decreased both embryonic and placental weights, aligning with MR evidence linking elevated maternal circulating AA to miscarriage risk. Decidual AA exposure amplified pro-inflammatory macrophages (CD11c^+^), cytotoxic natural killer (NK) cells (NKp46^+^, IFN-γ^+^), and cytotoxic T lymphocytes (CTLs, TNF-α^+^), contrasting OA-driven expansion of M2-like macrophages (CD206^+^) and pregnancy-protective NK cells (B220^+^CD11c^+^). Systemically, AA polarized Th1/CTL dominance (IFN-γ^+^CD8^+^) and Ly-6C^high^ monocyte retention, whereas OA enhanced Th2 responses and Ly-6C^low^ monocyte maturation. Paradoxically, AA up-regulated ULN tolerogenic dendritic cells (DCs) and IL-10 expressing regulatory B cells, suggesting tissue-specific lipid sensing. PA activated splenic IFN-γ^+^ NKs but spared decidual/ULN tolerance. In summary, distinct FAs differentially program gestational immunity in a tissue-specific manner: OA enforces systemic tolerance, while AA drives localized inflammation despite compensatory ULN immunosuppression. These findings advocate personalized FA interventions to optimize pregnancy outcomes.

## Introduction

Miscarriage is usually defined as the loss of an intrauterine pregnancy before the fetus reaches viability, up to 28 weeks of gestation [1]. Ulteriorly, two or more miscarriages are classified as recurrent spontaneous miscarriage (RSA), a condition that affects 1–2% of couples and imposes substantial long-term health and psychological burdens through its associated complications [2]. However, the etiology of RSA patients is multifactorial, comprising genetic, immunological, endocrine, and uterine factors [3]. To obtain a better understanding of miscarriage, research must extend beyond clinical observations, with suitable animal models being essential for elucidating its underlying progress. In pregnancy models, mice are widely used due to their large litter size, short generation periods, and low cost [4]. Meanwhile, mouse decidua exhibits intrinsic immunological features and spatiotemporally organized immune cell patterns that closely resemble those of human decidua [5]. Admittedly, mice differ from humans in placental development and gestation duration, which limits the direct clinical applicability [6]. Nevertheless, given their advantages, mice were selected for the present study to best mimic the pregnancy process.

To accommodate fetal growth and development, pregnant women experience physiological metabolic adaptations centered on changes in lipid metabolism [7]. During early-to-mid gestation, maternal tissues exhibit increased lipid deposition, which progressively transitions to a catabolic state in late pregnancy, characterized by physiological hyperlipidemia and elevated free fatty acids (FAs) [8]. However, when dyslipidemia occurs, it may impair endometrial receptivity, leading to embryo implantation failure and an increased risk of miscarriage [9]. Our previous study found that, compared with normal pregnancies, lipid accumulation was significantly increased in the decidua and villous tissues of patients who experienced miscarriage [10]. Additionally, Guleken et al. reported that patients with RSA also exhibit dysregulated maternal serum lipid profiles [11]. Hence, maintaining lipid metabolic homeostasis is fundamental for pregnancy. Emerging evidence suggests that alterations in FA levels are associated with pregnancy losses. For instance, women who experience miscarriage often exhibit perturbations in FA profile [12], and elevated levels of polyunsaturated fatty acids (PUFAs) in preconception plasma have been linked to an increased risk of miscarriage [13,14]. Of note, a decreased concentration of total monounsaturated fatty acids (MUFAs) has been observed in the villous tissues of RSA patients [15], suggesting that the heterogeneity of FAs may contribute to distinct pregnancy outcomes.

Maternal–fetal immune tolerance represents a finely balanced state in which the maternal immune system accepts the semi-allogenic fetus without compromising host defense [16,17]. This tolerance primarily relies on the dynamic remodeling of the immune microenvironment at the maternal–fetal interface, driven by adaptive phenotypic and functional changes in immune cells. Extensive studies have explored how FAs modulate immune cell phenotypes and functions [18–20]. Yet, how distinct FAs participate in and remodel maternal–fetal immune tolerance and how FA-induced immunometabolic changes affect pregnancy outcomes remain largely unexplored. This critical knowledge gap prompted us to conduct the present study.

In the present study, we employed *in vivo* mouse models to investigate the effects of FAs on pregnancy outcomes and maternal immune responses. We selected the three most abundant FAs in humans: saturated palmitic acid (PA), monounsaturated oleic acid (OA), and polyunsaturated arachidonic acid (AA) [21]. By analyzing immune cell phenotypes across three key maternal immune compartments (decidua – maternal–fetal interface; uterine-draining lymph node [ULN] and spleen – secondary lymphoid organs; maternal peripheral blood – circulating immune compartment), we extensively elucidated the distinct immunomodulatory roles of PA, OA, and AA. Mendelian randomization (MR) analysis was employed to define the potential causal relationship between specific FAs and spontaneous miscarriage. Our findings reveal an unprecedented interaction between nutritional metabolites and gestational immunity, providing a theoretical basis for optimizing dietary fat intake during pregnancy.

## Methods

### Animal models

Specific pathogen-free (SPF)-grade female and male C57BL/6 mice (6–8 weeks of age) were obtained from Shanghai JieSiJie Laboratory Animal Co. Ltd under controlled housing conditions (12 h light/dark cycle, 24 ± 1°C). Animals received a standard irradiated laboratory rodent breeding diet (Jiangsu Xietong Pharmaceutical Bio-Engineering Co. Ltd.) and water freely. The maintenance of the mice was conducted at the Laboratory Animal Center of Shanghai First Maternity and Infant Hospital (Shanghai, China). For pregnancy establishment, female mice were mated with fertile males. Vaginal plug observation was conducted each morning, with plug-positive females designated at gestational day 0.5 (GD0.5).

**Figure 1:**
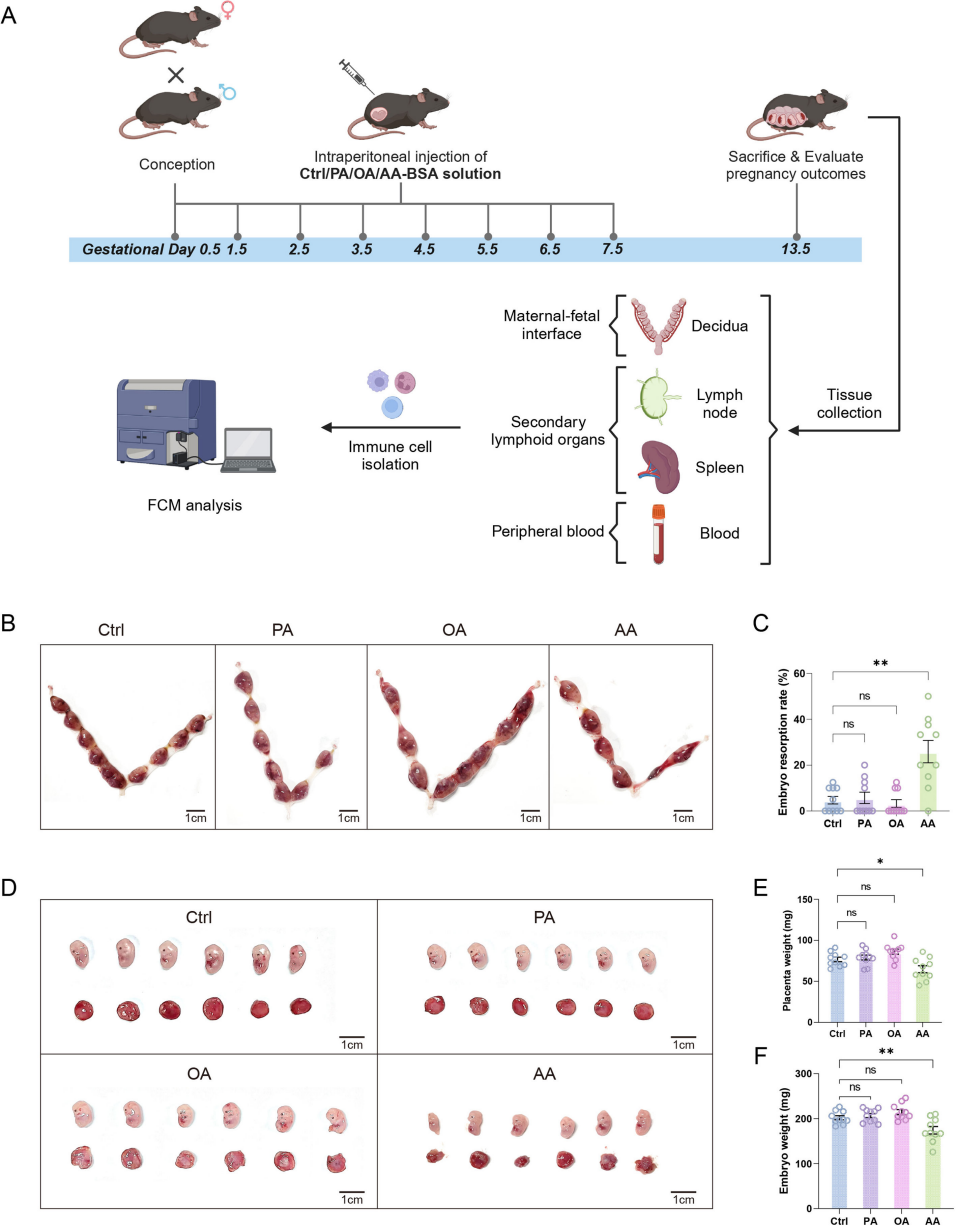
Arachidonic acid (AA) disrupts pregnancy outcomes in murine models (**A**) Schematic representation of the experimental workflow in mouse pregnancy models, encompassing model establishment, pregnancy outcome assessment, immune cell isolation, and flow cytometry (FCM) analysis across tissues. (**B**) Representative images of uteri in pregnant mice intraperitoneally administrated with different fatty acids (Ctrl/PA/OA/AA). (**C**) Statistical analyses of the embryo absorption rate in each group. (**D**) Representative images of embryos and placentae in pregnant mice intraperitoneally administrated with different fatty acids (Ctrl/PA/OA/AA). The weights of placentae (**E**) and embryos (**F**) were compared. Data are presented as mean ± SEM. Each symbol represents one biological replicate (individual mouse). NS, not significant; *P < 0.05; **P < 0.01.

****The use of dietary supplementation to study FAs presents a limitation, as it is difficult to ensure that specific FAs alter serum levels without affecting other lipid profiles following intestinal absorption and hepatic metabolism [22,23]. To overcome this limitation and more accurately elucidate the *in vivo* effects of specific FAs, we administered FAs via intraperitoneal injection in the form of bovine serum albumin (BSA) conjugates. Pregnant mice were randomly allocated to four groups receiving daily intraperitoneal administrations of 200 μL BSA-conjugated PA, OA, AA solutions or unloaded BSA as control at a concentration of 50 μM for 7 consecutive days. The concentration of 50 μM was selected based on previous *in vivo* and *in vitro* studies, demonstrating that it effectively modulates immune cell function without inducing lipotoxicity or acute stress responses [24,25]. This concentration is substantially lower than the physiological plasma levels of these FAs, which can reach up to 1000 μM for PA and OA and 300 μM for AA in humans [21], ensuring that our experimental approach probes specific immunomodulatory pathways rather than overwhelming the system. On GD13.5, mice were killed via cervical dislocation for collection of decidual tissues, peripheral blood, spleen, and ULNs, with embryo resorption rates quantified to assess pregnancy outcomes. Tissue-resident immune cells were isolated and examined by flow cytometry analysis (Figure 1A). All surgical and terminal procedures were performed under anesthesia induced by intraperitoneal injection of tribromoethanol (1.25%, 0.2 mL/10 g; Aladdin, China). Depth of anesthesia was confirmed by the absence of pedal reflex prior to blood collection. Post-procedure, no animals regained consciousness as euthanasia was performed via cervical dislocation under sustained anesthesia. All animal experiments were performed in compliance with the Chinese Guidelines for the Care and Use of Laboratory Animals and approved by the Institutional Animal Ethics Committee of Tongji University.

### FA–BSA conjugate synthesis

FA-enriched media were generated through sodium palmitate/oleate/arachidonate (PA/OA/AA) complexation with BSA, using a method slightly modified from the described protocol [26]. PA, OA, AA, and BSA were purchased from Sigma-Aldrich (St. Louis, MO, U.S.A.). Specifically, sodium FA salts were dissolved in 0.1 M NaOH pre-warmed to 75°C, followed by sequential incorporation into 30% (w/v) BSA solution maintained at 55°C under continuous agitation (30 min) to achieve stable FA–BSA conjugates. Post-sterilization through 0.45 μm filtration, aliquots were cryopreserved (–20°C) and thermally equilibrated (55°C, 10 min) prior to experimental application. Parallel control formulations contained equivalent BSA–NaOH mixtures devoid of lipids. Final working concentrations were adjusted to 50 μM through serial dilution in serum-free EMEM [27].

### Decidual immune cells and peripheral blood mononuclear cells isolation

Uterine tissues from GD13.5 mice were dissected under sterile conditions, with fetal and placental remnants removed via phosphate-buffered saline (PBS) irrigation. Mechanically fragmented specimens underwent enzymatic dissociation in RPMI 1640 medium (Gibco, U.S.A.) containing collagenase type IV (1 mg/mL; Sigma-Aldrich) and DNase I (200 U/mL; Sigma-Aldrich), followed by orbital agitation (37°C, 30 min). The resulting suspension underwent sequential filtration through 70 μm nylon mesh prior to density-based stratification using a discontinuous Percoll gradient (GE Healthcare, U.S.A.). Decidual immune cell (DIC) populations were selectively recovered from the bottom second layer. Peripheral blood underwent 1:1 volumetric dilution in PBS and stratified over equal-volume Ficoll medium. Following centrifugation (800×g, 20 min), peripheral blood mononuclear cells (PBMCs) were harvested from the plasma–Ficoll interface.

### Secondary lymphoid tissue dissociation

Murine spleens were aseptically harvested into RPMI 1640 medium and mechanically dissociated through 70 μm nylon mesh using plunger-assisted filtration. Single-cell suspensions were maintained in complete RPMI 1640 (10% FBS, 100 U/mL penicillin, 100 μg/mL streptomycin, 1 μg/mL amphotericin B) at 37°C/5% CO_2_. ULNs underwent equivalent sterile processing with 40 m mesh filtration. Post-PBS rinsing, cells were cultured under identical medium conditions.

### Flow cytometry

Cellular protein profiling was performed using a flow cytometer (Beckman Coulter, CyAN ADP analyzer) following standardized immunolabeling procedures. Single-cell suspensions (1 × 10⁶ cells/sample) were pre-treated with mouse-specific Fc receptor blocking reagents to minimize nonspecific binding. Surface marker profiling utilized fluorochrome-conjugated antibodies (PE-conjugated anti-mouse/human CD4, BV421-conjugated anti-mouse CD3, APC-conjugated anti-mouse CD4, PerCP-conjugated anti-mouse CD8, BV785-conjugated anti-mouse F4/80, AF700-conjugated anti-mouse NK1.1, PE-Cyanine 7-conjugated anti-mouse major histocompatibility complex II [MHC-II], APC-conjugated anti-mouse CD11c, BV421-conjugated anti-mouse CD11b, PE-conjugated anti-mouse B220, FITC-conjugated anti-mouse NKp46, FITC-conjugated anti-mouse PDCA-1, APC-conjugated anti-mouse Ly-6C, FITC-conjugated anti-mouse CD19, PerCP-conjugated anti-mouse CD86, and PE/Dazzle™ 594-conjugated anti-mouse CD206) during 30 min 4°C incubation. For intracellular targets, cells were processed via Fix/Perm kit (BioLegend) per the manufacturer’s guidelines before staining with FITC-conjugated anti-mouse TNF-α, PE-Cyanine 7-conjugated anti-mouse IFN-γ, BV605-conjugated anti-mouse IL-4, and PE/Dazzle™ 594-conjugated anti-mouse IL-10 (30 min, 4°C dark). All experimental data underwent computational processing through specialized analysis software (FlowJo, v10.8.1).

### MR analysis

Two-sample MR analysis uses single-nucleotide polymorphisms (SNPs) as instrumental variables (IVs) to explore the potential causal association between specific FAs (AA, OA, and PA) and miscarriages (number of spontaneous miscarriages and recurrent spontaneous miscarriage). Genome-wide association study (GWAS) summary statistics included in the present study were downloaded from PubMed, EBI GWAS catalog, and FinnGen database (Supplementary File 1). MR analysis relies on three key assumptions [28]: (1) a direct correlation of SNPs with the FA exposures, (2) the SNPs’ lack of connection to any confounders, and (3) SNPs are not associated with outcomes except through the exposure. Specifically, we initially selected SNPs based on associations with circulating blood levels of FAs, as derived from GWAS summary statistics (significance level set at 1 × 10^−5^). To address linkage disequilibrium, we performed clumping with *r* ^2^ < 0.001 and clumping distance = 10,000 kb [29,30]. Following the exclusivity assumption, we excluded SNPs’ significant association with outcomes (*P*<5 × 10^−5^). Furthermore, we utilized Steiger filtering and harmonized the data to remove SNPs that did not meet the criteria [31,32]. We also calculated the *F*-statistic by the following formula to assess the strength of SNPs, where *F* > 10 indicated sufficient (Supplementary File 2) [33].


$$F=\frac{R^{2}\left(N-1-K\right)}{\left(1-R^{2}\right)K}$$

$$R^{2}=2\times MAF\times \left(1-MAF\right)\times beta^{2}$$

*N*: number of individuals, *K*: number of variants involved in IV model, *R* ^2^: variance explained by IVs, EAF: effect allele frequency, MAF: minor allele frequency.

We then scrutinized the SNPs for associated phenotypes in the NHGRI-EBI Catalog to better meet the independence assumption (Supplementary File 3) [34]. We employed multiple methods to estimate causal effects, including inverse-variance weighted (IVW) [35], weighted median [36], weighted mode [37], MR-Egger regression [38], and MR Pleiotropy Residual Sum and Outlier (MR-PRESSO) [39]. Sensitivity analysis was conducted for check robustness: (1) Heterogeneity was assessed through IVW and MR-Egger [40]. (2) Horizontal pleiotropy was evaluated through MR-Egger and MR-PRESSO [39,41,42] (Supplementary File 4). In MR analysis, the combined β-values and scatterplot can indicate the strength of the association between the exposure and outcome, while OR values and *P* values provide statistical evidence for the evaluation of potential causal relationships. All analyses were conducted using R software (v4.1.2), leveraging the TwoSampleMR (v0.5.8), MendelianRandomization (v0.8.0), and MR-PRESSO (v1.0) packages. A two-sided *P* value < 0.05 was considered statistically significant.

### Statistical analysis

Data are presented as mean ± SEM and analyzed using GraphPad Prism 8.0. Normality distribution was verified through Shapiro–Wilk testing prior to selecting parametric (one-way ANOVA) or nonparametric (Kruskal–Wallis) approaches for multigroup comparisons, with Bonferroni *post hoc* adjustments. Categorical variables underwent χ² analysis. A probability threshold of *P*<0.05 defined statistical significance.

## Results

### General effects of PA, OA, and AA on pregnancy outcomes

To detect the effects of different FAs on pregnancy outcomes and maternal immune responses, we established three FA intraperitoneal injection mouse models using BSA-conjugated PA, OA, or AA solutions with unloaded BSA as control. The immune cell markers, their associated cell types, and their indicative effects on the functional phenotype of specific immune cells were summarized in Supplementary File 5. Compared with the control group, either PA or OA had no apparent effect on the embryo resorption rate, whereas AA significantly induced abortions (Figure 1B and C). Similarly, AA remarkably reduced the weights of both the embryo and the placenta, but no effect was observed with PA and OA (Figure 1D–F). Therefore, AA, but not PA or OA, is detrimental to pregnancy outcomes.

### OA and AA exert opposite effects on decidual innate immune cells

**Figure 2:**
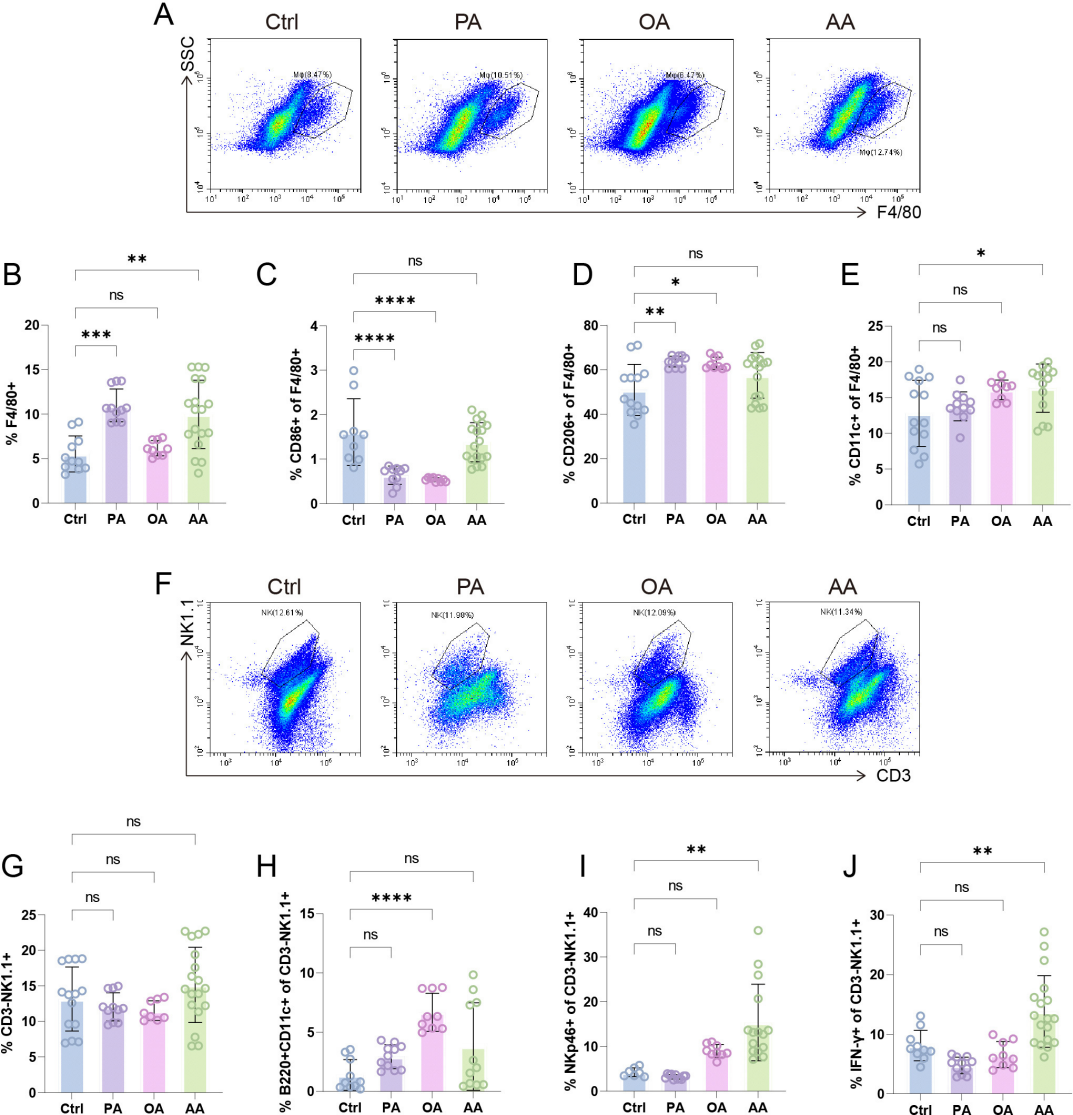
Distinct fatty acids (FAs) differentially modulate the phenotypes of decidual innate immune cells. (**A**) Flow cytometry (FCM) analysis of surface markers in F4/80^+^ decidual macrophages. (**B**) Proportions of F4/80^+^ cells in decidual immune cells (DICs), with further analysis of CD86^+^ (**C**), CD206^+^ (**D**), and CD11c^+^ (**E**) subpopulations within F4/80^+^ macrophages. (**F**) Characterization of natural killer (NK) cell subset heterogeneity in murine decidua. Statistical analyses of CD3⁻NK1.1^+^ bulk population within DIC (**G**), followed by B220^+^CD11c^+^ dual-positive (**H**), NKp46^+^ single-positive (**I**), and IFN-γ^+^ single-positive (**J**) NK cell percentages. Data are presented as mean ± SEM. Each symbol represents one biological replicate. Exact sample sizes per group are indicated in the figure. Discrepancies in sample sizes between panels resulted from the exclusion of samples that did not meet quality control thresholds during FCM data acquisition. NS, not significant; *P < 0.05, **P < 0.01, ***P < 0.001, ****P < 0.0001.

Among DIC populations, macrophages and NK cells constitute the predominant innate immune components. We first detected the changes in decidual macrophages (dMφ). FCM results showed that PA and AA significantly increased F4/80^+^ dMφ proportions, while OA exerted no significant effect (Figure 2A and B). Both PA and OA promoted M2-like immunosuppression while suppressing M1-like activation (Figure 2C and D and Supplementary Figure 1A). In contrast, AA showed no modulatory capacity in this axis (Figure 2C and D and Supplementary Figure 1A. The CD11c^+^ dMφ subpopulation, associated with pro-inflammatory and anti-angiogenic functions [43,44], was selectively expanded by AA but remained unaltered by PA or OA (Figure 2E and Supplementary Figure 1B). Taken together, PA and OA synergistically enhance tolerance-promoting dMφ profiles, whereas AA drives pro-inflammatory dMφ activation.

 Notably, none of the tested FAs (PA, OA, or AA) altered the total decidual NK (dNK) cell quantity (Figure 2F and G). Human dNK cells are characterized by a unique CD56^bright^CD16^dim^ immunophenotype [45]. Previous studies have established that murine B220^+^CD11c^+^NK1.1^+^ dNK cells represent the functional homolog of human CD56^bright^ dNK cells [46,47]. Our phenotypic analysis revealed that OA selectively enhanced the B220^+^CD11c^+^CD3^–^NK1.1^+^ dNK subpopulation, whereas PA and AA showed no significant effects (Figure 2H and Supplementary Figure 1C). AA specifically up-regulated the activation marker NKp46 and inflammatory cytokine IFN-γ production in dNK cells, but PA and OA had no effect (Figure 2I and J and Supplementary Figure 1D and E). This functional dichotomy demonstrates that OA promotes pregnancy-compatible dNK specialization, while AA drives pathological cytotoxic activation – suggesting opposing immunoregulatory roles of these FAs in gestational NK cell programming.

### AA impedes decidual T cell immunosuppressive bias

**Figure 3:**
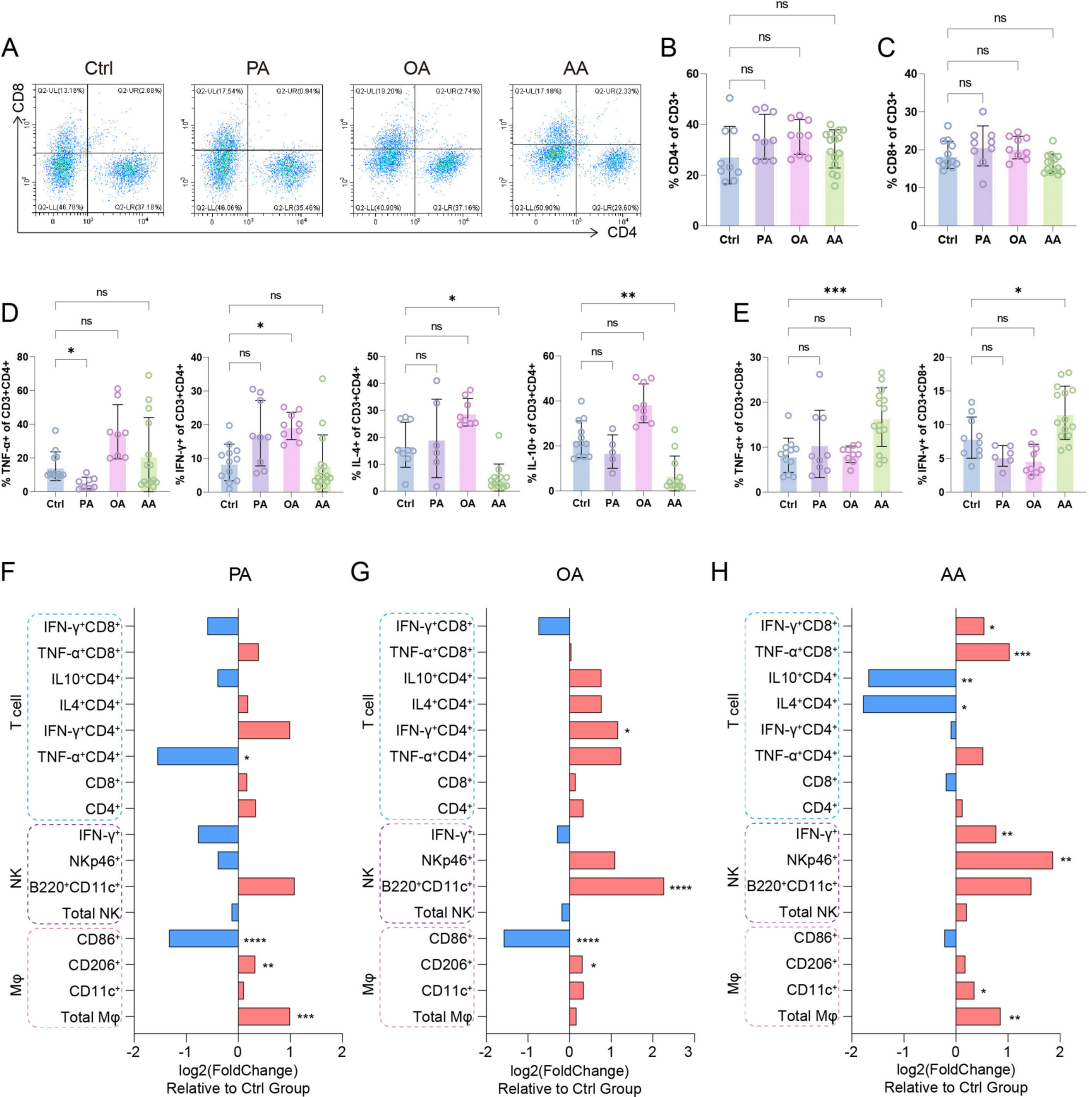
Fatty acid (FA)-driven reprogramming of decidual T cell immunity (**A**) Representative plots of decidual CD4^+^/CD8^+^ T cells. Proportions of total decidual CD4^+^ (**B**) and CD8^+^ T cells (**C**). (**D**) CD4^+^ functional profiles: frequencies of Th1 (IFN-γ^+^/TNF-α^+^) and Th2 (IL-4^+^/IL-10^+^) subsets. (**E**) CD8^+^ cytotoxic profiling: proportions of TNF-α^+^ and IFN-γ^+^ effector subpopulations. (**F–H**) Summaries of fatty acid-classified immunomodulation: Log2 fold-changes of the phenotypic markers across decidual T cells, NK cells, and macrophages in pregnant mice treated with PA (**F**), OA (**G**), or AA (**H**). Heatmap-style bars indicate up-regulation (red) or down-regulation (blue) versus control. Data are presented as mean ± SEM. Each symbol represents one biological replicate. Exact sample sizes per group are indicated in the figure. Discrepancies in sample sizes between panels resulted from the exclusion of samples that did not meet quality control thresholds during flow cytometry (FCM) data acquisition. NS, not significant; *P < 0.05, **P < 0.01, ***P < 0.001, ****P < 0.0001.

In the analysis of decidual T cells, the dominant adaptive immune constituents at the maternal–fetal interface, we found that while PA, OA, and AA did not alter total CD4^+^ or CD8^+^ T cell frequencies (Figure 3A–C), they induced functionally divergent cytokine polarization. PA suppressed pro-inflammatory cytokine TNF-α production while OA enhanced IFN-γ expression in CD4^+^ T cells (Figure 3D). AA selectively down-regulated immunosuppressive cytokines IL-4 and IL-10, shifting Th1/Th2 balance toward inflammatory bias (Figure 3D). In CD8^+^ T cells, AA uniquely amplified cytotoxic effector populations, elevating both TNF-α^+^ and IFN-γ^+^ subsets, whereas PA/OA showed no significant immunomodulatory effects (Figure 3E). This FA-specific reprogramming reveals that AA dually disrupts decidual tolerance by attenuating Th2-mediated immunosuppression and potentiating cytotoxic CD8^+^ T cell responses. Collectively, these findings position AA as a critical regulator of T cell-dependent immune homeostasis breach at the maternal–fetal interface.

 Our comprehensive profiling of DICs reveals distinct immunomodulatory hierarchies among FAs: saturated PA predominantly drives anti-inflammatory polarization in dMφ through M2-dominant reprogramming (Figure 3F), while monounsaturated OA co-ordinately enhances innate immune tolerance by expanding both M2-like dMφ and pregnancy-protective B220^+^CD11c^+^ dNK subsets (Figure 3G). Strikingly, AA emerges as a pan-immunological disruptor, simultaneously priming pro-inflammatory dMφ, amplifying cytotoxic dNK cells, and subverting T cell tolerance through Th2 suppression and cytotoxic T lymphocyte (CTL) activation – collectively destabilizing the immunotolerant microenvironment (Figure 3H). This FA-classified immunoregulatory paradigm positions nutritional lipids as master switches of gestational immunity, where ω-9 MUFA (OA) exerts protective effects, while ω-6 PUFA (AA) emerges as potent disruptor of decidual immune homeostasis.

### OA and AA induce tolerogenic immune responses in ULNs

**Figure 4:**
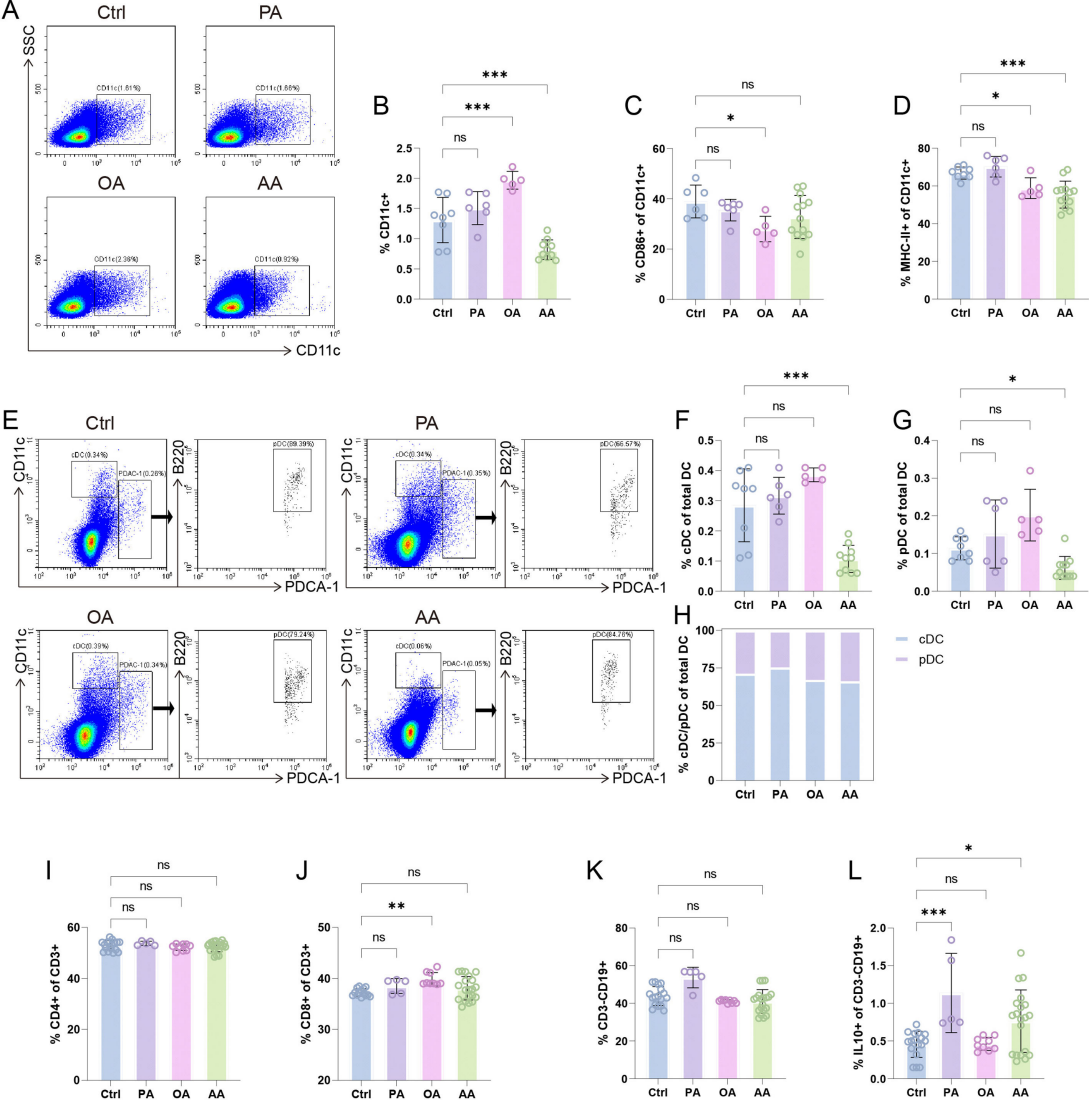
Oleic acid (OA) and arachidonic acid (AA) redirect uterine-draining lymph nodes (ULNs) toward tolerogenic microenvironment. (**A**) Representative flow cytometry (FCM) plots of CD11c^+^ dendritic cells (DCs). Statistical analyses of total DC numbers in ULNs (**B**), CD86^+^ (**C**), and MHC-II^+^ (**D**) subset percentages in DCs. (**E**) Gating strategy of conventional DC (cDC) (CD11c^+^PDCA-1⁻) and pDC (B220^+^PDCA-1^+^) subpopulations in CD11c^+^ DCs. Quantified frequencies of cDC (**F**), plasmacytoid DC (pDC) (**G**), and cDC/pDC composition (**H**) within total DC compartment. Frequencies of CD4^+^ (**I**) and CD8^+^ (**J**) T cells in ULNs. Statistical analyses of total CD3⁻CD19^+^ B cells (**K**) and IL-10^+^ regulatory B cells (**L**) in ULNs. Data are presented as mean ± SEM. Each symbol represents one biological replicate. Exact sample sizes per group are indicated in the figure. Discrepancies in sample sizes between panels resulted from the exclusion of samples that did not meet quality control thresholds during FCM data acquisition. NS, not significant; *P < 0.05, **P < 0.01, ***P < 0.001.

ULNs serve as a secondary maternal–fetal interface during pregnancy [48]. Our findings demonstrate that OA significantly increased total DC numbers in ULNs, whereas AA caused marked DC depletion, with PA showing no discernible effect (Figure 4A and B). Because mature DCs efficiently prime T cells and immature DCs promote antigen-specific T cell tolerance, we assessed DC maturation in ULNs by measuring CD86 and MHC-II expression. OA treatment down-regulated both CD86 and MHC-II, whereas AA selectively inhibited MHC-II (Figure 4C and D and Supplementary Figure 2A and B).

To dissect these effects further, we quantified conventional DCs (cDCs; CD11c^+^) and plasmacytoid DCs (pDCs; CD11c^low^ PDCA-1^+^ B220^+^). cDCs serve as the most potent professional antigen-presenting cells (APCs), priming naïve T cells and initiating adaptive immune responses. In contrast, pDCs exhibit limited immunostimulatory capacity under steady state but are adept at inducing and expanding regulatory T cells [49]. FCM revealed that OA and PA did not alter the absolute counts of either subset, while AA significantly reduced both cDCs and pDCs, consistent with the overall DC decline in the AA group (Figure 4E–G). Additionally, the cDC/pDC ratio increased under PA but decreased with both OA and AA (Figure 4H). Together, these findings demonstrate that both OA and AA foster a tolerogenic DC milieu in ULNs – OA primarily by inducing an immunosuppressive phenotype (maturation arrest and pDC preservation), and AA chiefly by reducing DC abundance – thereby sculpting an immunoregulatory niche through complementary mechanisms.

We next assessed adaptive immune populations within ULNs. As shown in Figure 4I and J and Supplementary Figure 2C, CD4^+^ T cell numbers remained comparable across all groups, while OA selectively expanded the CD8^+^ T cell compartment. Total B cell counts were likewise unchanged; however, both PA and AA significantly increased the fraction of regulatory B cells (Bregs) – critical contributors to pregnancy tolerance (Figure 4K and L and Supplementary Figure 2D and E) [50,51]. These data indicate that AA exerts a unique modulatory effect in ULNs, driving tolerogenic adaptive responses rather than pro-inflammatory activation.

### Distinct effects of PA, OA, and AA on splenic and peripheral NK and T cells

**Figure 5:**
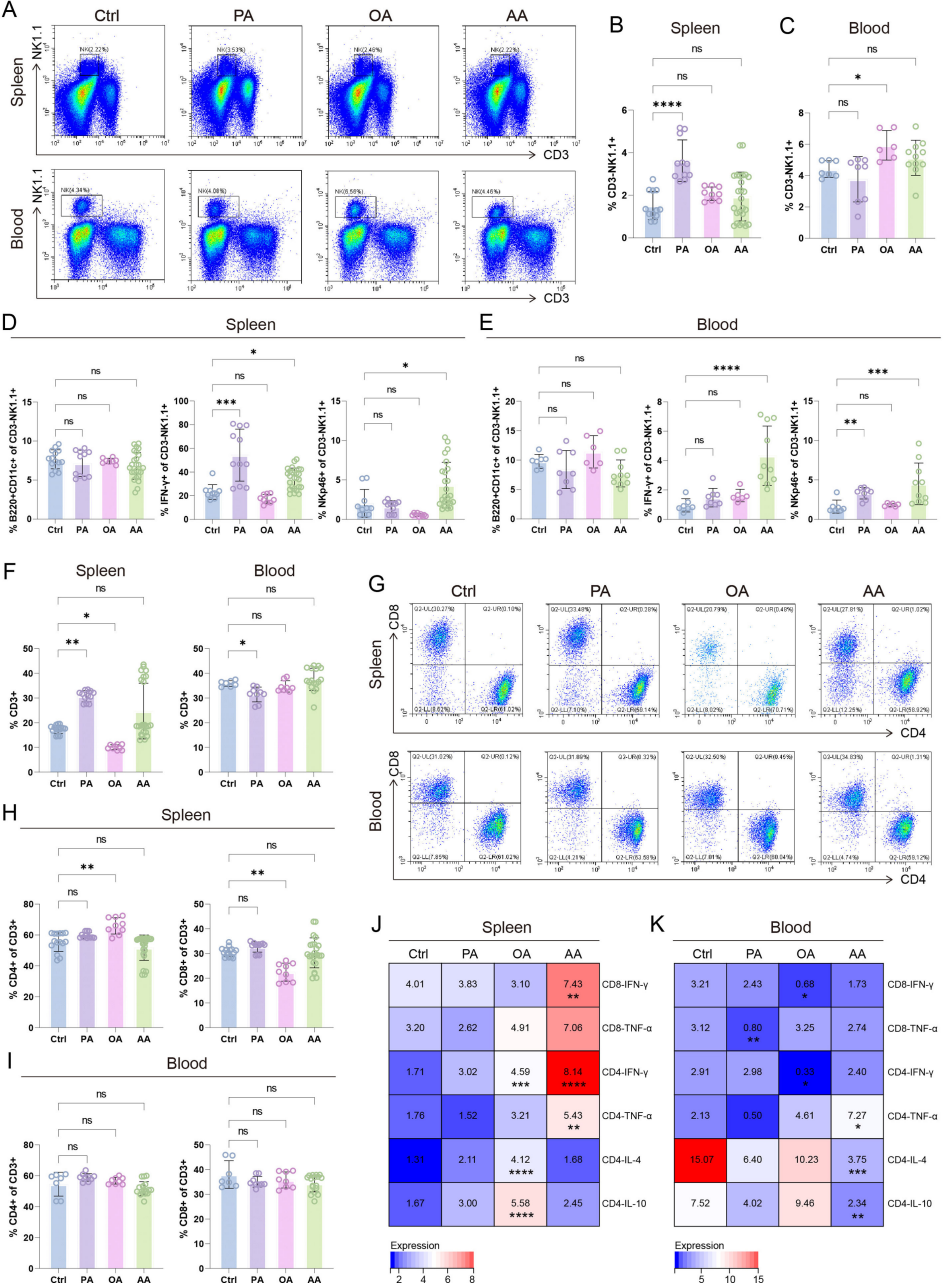
Fatty acid (FA)-classified immunomodulation in splenic and peripheral natural killer (NK)/T cell compartments (**A–C**) Representative flow cytometry (FCM) plots and quantitative proportions of NK cell in splenic and peripheral blood across different groups. Statistical analyses of B220^+^CD11c^+^ (left), IFN-γ^+^ (middle), and NKp46^+^ (right) subsets in splenic (**D**) and blood (**E**) NK cells. Quantification of total T cells (**F**), CD4^+^ and CD8^+^ T cell subsets (**G**) in splenic (**H**) and peripheral blood (**I**) compartments. Heatmap of cytokine expression intensities (IFN-γ, TNF-α, IL-4, IL-10) in CD4^+^ and CD8^+^ T cells in spleen (**J**) and peripheral blood (**K**). Data are presented as mean ± SEM. Each symbol represents one biological replicate. Exact sample sizes per group are indicated in the figure. Discrepancies in sample sizes between panels resulted from the exclusion of samples that did not meet quality control thresholds during FCM data acquisition. NS, not significant; *P < 0.05, **P < 0.01, ***P < 0.001, ****P < 0.0001.

We then extended our analysis to pregnancy-driven systemic immunity by profiling maternal splenic and peripheral immune cell functions. As shown in Figure 5A–C, PA selectively elevated the frequency of NK cells in the spleen, whereas OA specifically expanded the NK cell pool in peripheral blood. The subset of B220^+^CD11c^+^ NK cells, whose presence is linked to pregnancy tolerance, remained constant across all groups in both compartments (Figure 5D and E). In the spleen, PA and AA both up-regulated IFN-γ production in NK cells, while only AA significantly increased NKp46 expression (Figure 5D). A parallel trend was observed in peripheral NK cells: AA robustly boosted the proportions of IFNγ^+^ and NKp46^+^ cells, and PA also enhanced NKp46 levels (Figure 5E). Collectively, these findings demonstrate that PA and AA activate NK cells systemically, contributing to the modulation of maternal innate immunity during pregnancy.

 We next evaluated T cell responses systemically. Interestingly, PA increased total T cell numbers in the spleen but decreased circulating T lymphocyte counts (Figure 5F). In contrast, OA selectively decreased splenic T cells, whereas AA had no significant impact in either compartment (Figure 5F). Subset analysis in the spleen showed that OA alone elevated CD4^+^ T cells while reducing CD8^+^ T cells (Figure 5G and H), yet none of the FAs altered the CD4^+^ or CD8^+^ ratio in peripheral blood (Figure 5G and I). Functionally, OA enhanced IFN-γ, IL-4, and IL-10 production in splenic CD4^+^ T cells, whereas AA selectively boosted IFN-γ in CD8^+^ T cells and both IFN-γ and TNF-α in CD4^+^ T cells (Figure 5J). In peripheral blood, OA concurrently suppressed IFN-γ expression in both CD8^+^ and CD4^+^ T cells (Figure 5K), while AA up-regulated the Th1 cytokine TNF-α and down-regulated Th2 cytokines IL-4 and IL-10 (Figure 5K). Little phenotypic or functional changes were observed in T cells from PA-treated mice (Figure 5J and K). Collectively, this dichotomous regulation establishes OA as a systemic immunosuppressive modulator and AA as a multitiered inflammatory driver, highlighting their opposing roles in shaping the T cell cytokine milieu.

### PA and AA activate, OA inhibits monocyte–macrophage inflammatory features

**Figure 6:**
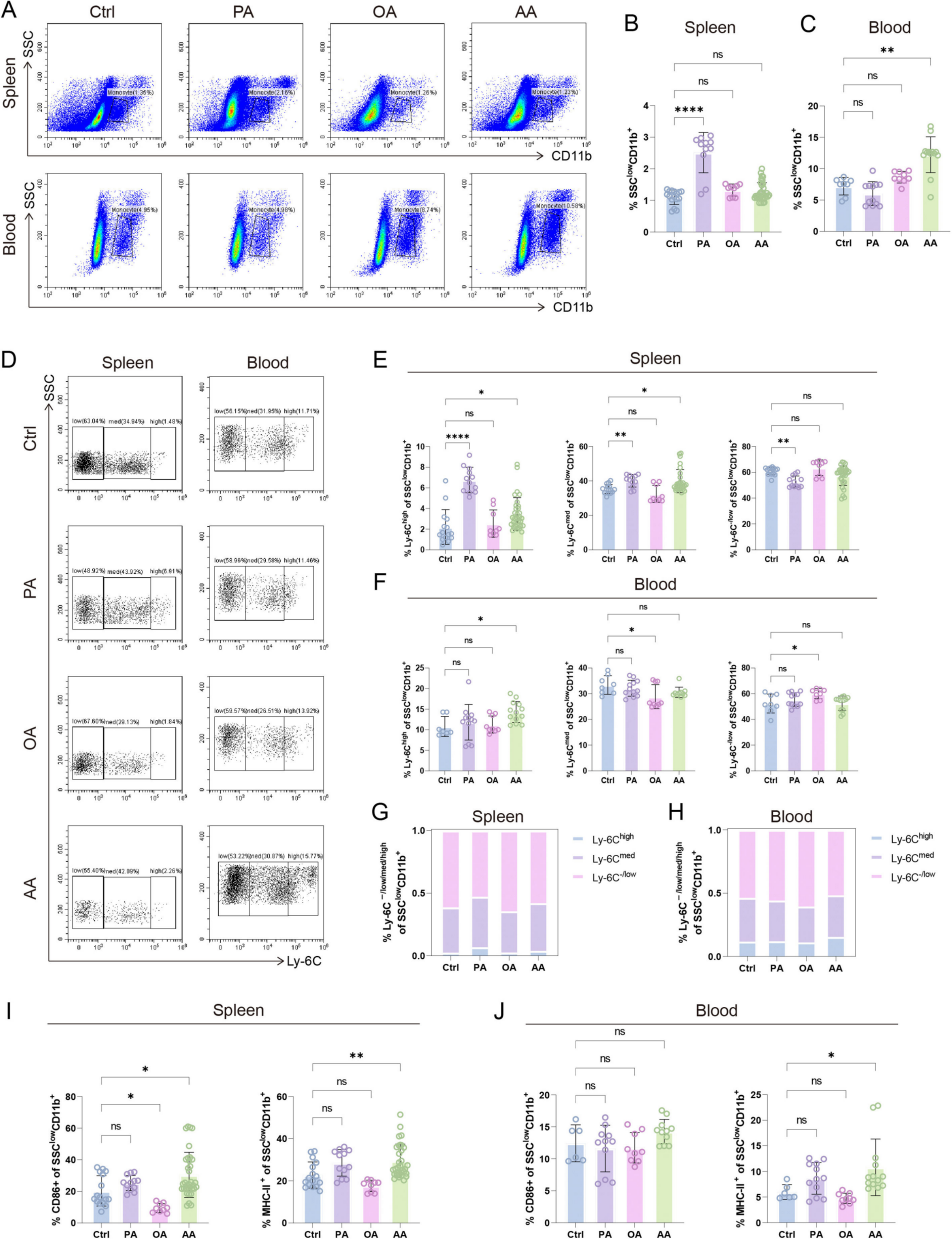
Effects of different fatty acids (FAs) on splenic and peripheral monocyte–macrophage systems (**A–C**) Flow cytometry (FCM) quantification of SSC^low^CD11b^high^ monocyte–macrophage frequencies in splenic and peripheral compartments. (**D–F**) Proportional distribution of monocyte subsets stratified by Ly-6C expression tiers (high/intermediate/low) within total SSC^low^CD11b^high^ monocytes. The classified Ly-6C (low/intermediate/high) distribution within total monocytes compartment in spleen (**G**) and peripheral blood (**H**). Ratios of CD86^+^ and MHC-II^+^ subsets in monocyte–macrophages in spleen (**I**) and peripheral blood (**J**). Data are presented as mean ± SEM. Each symbol represents one biological replicate. Exact sample sizes per group are indicated in the figure. Discrepancies in sample sizes between panels resulted from the exclusion of samples that did not meet quality control thresholds during FCM data acquisition. NS, not significant; *P < 0.05, **P < 0.01, ****P < 0.0001.

The monocyte–macrophage system orchestrates immune surveillance through pathogen clearance, antigen presentation, and cytokine regulation. Our analysis revealed FA-specific modulation of this system: PA selectively expanded splenic CD11b^+^ monocyte–macrophages, while AA augmented peripheral CD11b^+^ populations, with OA showing no quantitative effects (Figure 6A–C). Monocyte maturation, marked by the down-regulation of Ly-6C expression and the weakening of inflammatory phenotypes [52], exhibited anatomical dichotomy – PA retained splenic monocytes in immature Ly-6C^high/med^ states while suppressing mature Ly-6C^–/low^ subsets, whereas OA promoted peripheral monocyte maturation through Ly-6C down-regulation (Figure 6D–H). Conversely, AA sustained inflammatory Ly-6C^high/med^ dominance across both compartments (Figure 6D–H), indicative of systemic maturation arrest.

Functional profiling demonstrated that OA attenuated splenic CD86^+^ monocyte–macrophage activation, while AA robustly amplified inflammatory signatures through dual induction of CD86^+^ and MHC-II^+^ subsets (Figure 6I and J). This triple-axis regulation establishes PA and AA as potent drivers of monocyte–macrophage inflammation, contrasting with OA’s immunosuppressive reprogramming through maturation promotion and co-stimulatory molecule suppression.

### Insights into FAs and pregnancy outcomes from MR analysis

**Figure 7:**
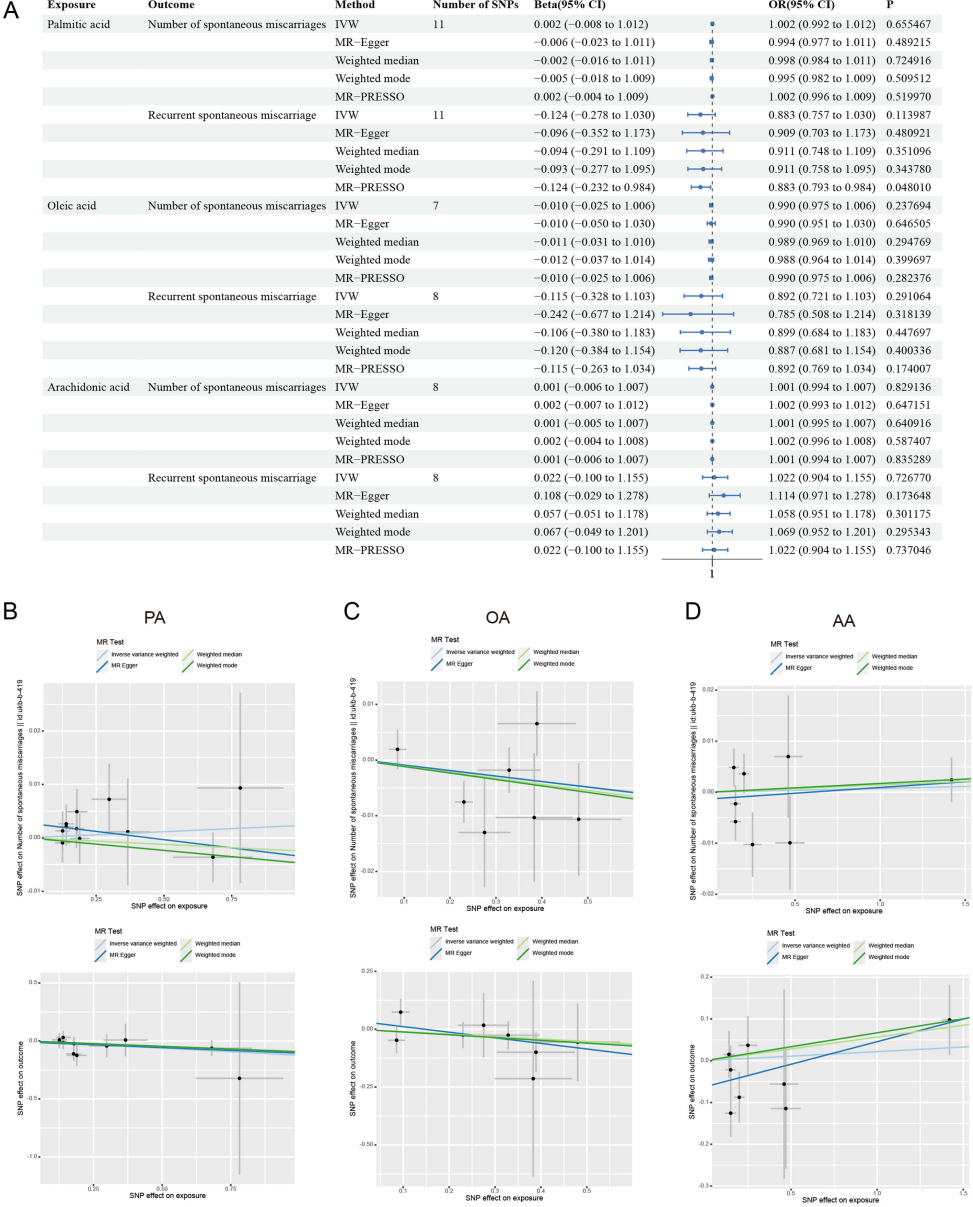
Causal links between specific fatty acids and miscarriages. (**A**) Forest plots showing Mendelian randomization (MR) results testing for causal relationships between fatty acids and miscarriages. Scatterplots depicting causal effects of fatty acids on miscarriages, explicated with the number of spontaneous miscarriages and recurrent spontaneous miscarriage risk across palmitic acid (PA) (**B**), oleic acid (OA) (**C**), and arachidonic acid (AA) (**D**)

Initial evidence has established that AA may induce miscarriage, while PA has no notable effect. Though OA did not significantly reduce resorption rates in our mouse model, it promoted tolerogenic immune phenotypes across multiple tissues, suggesting a protective immunomodulatory role. Focusing on PA, OA, and AA, we present a comprehensive MR analysis of the effects of specific FAs on miscarriages, including the number of spontaneous miscarriages and recurrent spontaneous miscarriage. As for PA, the inconsistency in β-values across methodologies when considering the number of miscarriages suggested an unstable effect. While PA showed an association with increased risk in recurrent miscarriages, a definitive causal relationship was not established (Figure 7A and B). The results for OA showed a protective effect against recurrent miscarriages. Unfortunately, despite the positive β-values, the lack of statistical significance ruled out the assertion of a causal relationship (Figure 7A and C). AA is associated with an increased risk of miscarriage, but a definitive causal link was not established (Figure 7A and D), suggesting that while there is a trend, it is not statistically significant.

## Discussion

Lipid homeostasis is a determinant of both maternal–fetal nutrition and pregnancy outcomes. In addition to serving as substrates for energy metabolism, FAs are important signaling molecules involved in maintaining the homeostasis of immune microenvironment by modulating the phenotype and function of immune cells. Our study systematically revealed FA-dependent maternal–fetal immunomodulation: (1) AA induced embryo resorption and decreased fetal and placental weights, correlating with systemic pro-inflammatory priming in decidua, spleen, and periphery. Paradoxically, AA enforced ULN immunosuppression via cDC/pDC ratio reduction, monocyte depletion, and Breg up-regulation; (2) OA systemically attenuated inflammation: down-regulating CD86^+^ and MHC-II^+^ monocytes across tissues while elevating anti-inflammatory IL-4^+^ and IL-10^+^ CD4^+^ T cells; (3) PA exhibited compartmentalized effects – enhancing splenic Ly6C^high^ monocytes and IFN-γ^+^ NKs without triggering decidual/ULN inflammation. This anatomical stratification of FA effects reveals an evolutionary trade-off between metabolic utilization and immune adaptation, where FA diversity enables spatiotemporal regulation of gestational immunity. Furthermore, the landmark MR analysis demonstrated a positive association between genetically predicted circulating AA levels and spontaneous abortion risk, contrasted by a protective inverse association for OA. This bidirectional genetic evidence establishes maternal lipid metabolism as a modifiable risk factor for pregnancy maintenance, providing genetic evidence for dietary lipid optimization in prenatal care.

 Decidua is the central maternal–fetal interface co-ordinating DIC interactions to serve as a physical barrier. Driven by the dominant immune tolerance in the second trimester [53], DICs acquire immunosuppressive phenotypes [54–56]. To systematically explore the effect of FAs on dNKs, we quantified NKp46, IFN-γ, and the co-expression patterns of B220 and CD11c. Previous studies have demonstrated a consistent up-regulation of NKp46 throughout normal pregnancy, contrasting with its diminished receptor expression observed in RSA patients [57–60]. Notably, our investigation regarded NKp46 as the canonical activating receptor of NK cells, wherein AA up-regulated the NKp46^+^ dNK proportion. This result can be explained by the differential expression patterns: NKp46 predominantly localizes to CD56^dim^CD16^–^ dNK subset in miscarriage, which is characterized by heightened cytotoxicity, in contrast with its predominant association with CD56^bright^CD16^–^ dNK subset during physiological gestation [61,62]. Additionally, emerging evidence classifies murine peripheral and uterine B220^+^CD11c^+^NK1.1^+^ cells within the NK cell lineage, which appear to have the functional convergence of human CD56^bright^CD16^–^ dNK subset [46,47]. Given the predominant B220 expression in most dNKs and its significant up-regulation in dNKs during gestation [47], coupled with our observation that OA enriched B220^+^CD11c^+^ dNK frequency, we postulate that OA may critically support physiological pregnancy maintenance. Concomitant up-regulation of IFN-γ and IL-4 in CD4^+^ T cells was observed in OA-treated groups. Although IFN-γ is regarded as a pro-inflammatory Th1 cytokine in gestation, it is paradoxically indispensable at low doses for angiogenic remodeling of uterine spiral arteries during implantation [63,64]. Hence, our data exhibited dual regulatory capacity of IFN-γ in pregnancy sustenance without challenging the Th2-skewed paradigm. Furthermore, at the maternal–fetal interface, AA selectively down-regulated the production of the critical anti-inflammatory cytokines IL-4 and IL-10 in decidual CD4^+^ T cells. This suppression of the Th2 cytokine milieu, which is essential for maintaining immune tolerance, represents a direct mechanistic pathway through which AA disrupts fetal–maternal immunotolerance and promotes a local inflammatory environment conducive to pregnancy failure.

Beyond the decidua, which serves as the primary immune interface at the maternal–fetal junction, we further identified similar immunological phenomena in the spleen and peripheral circulation, functioning as hubs for systematic immune co-ordination. We demonstrated that AA promoted pro-inflammatory phenotypes, including NKp46^+^ NK cells, IFN-γ^+^ NK cells, MHC-II^+^ monocytes, and CD86^+^ monocytes in both spleen and peripheral blood. Furthermore, we established Ly-6C maturation dynamics as a functional biomarker to interrogate monocyte migratory potential and tissue infiltration capacity at inflammatory foci. Sunderkötter et al. [52] delineated a monocyte maturation trajectory wherein Ly-6C^high^ precursors are released into peripheral circulation, undergoing progressive down-regulation to Ly-6C^low^ phenotypes during physiological maturation – a dynamic process reflecting their acquisition of tissue-adaptive immunoregulatory functions under homeostatic conditions. Whereas Ly6C^high^ monocytes are retained in tissues for a prolonged period when exposed to inflammation. Our research revealed that PA and AA can individually enhance the content of Ly6C^high^ monocyte–macrophages in spleen and peripheral blood. Meanwhile, OA up-regulated the fraction of Ly6C^low^ monocytes accompanied by reduced CD86^+^ counterparts. These experimental results collectively suggest that PA and AA promote local and systemic inflammatory responses in pregnant women, while OA exhibits partial anti-inflammatory effects that may improve pregnancy outcomes.

 DCs, one of the primary sentinels encountering fetal antigens, uniquely orchestrate the balance between the immune response and tolerance. Notably, patients suffering from pre-eclampsia and miscarriage showed elevated frequency of cDCs coupled with a diminished fraction of pDCs, prompting that cDC/pDC imbalance is associated with adverse pregnancy outcomes [65–67]. Moreover, Fang et al. demonstrated that the differentiation of cDCs and pDCs during pregnancy in the mouse exhibited tissue-specific characteristic, as manifested by a decreased cDC/pDC percentage exclusively in para-aortic lymph nodes without alterations in the spleen [49]. Strikingly, our data appear to indicate a contradictory conclusion in ULNs compared with decidua, spleen, and peripheral circulation. In the AA-treated group, the cDC/pDC ratio decreased relative to the controls, coinciding with a numerical reduction in both subsets. This paradoxical phenomenon also extended to IL-10-producing Bregs, which typically increase during early gestation to enforce decidual immunosuppression [68–70]. Although a reduction in peripheral Bregs was observed in RSA patients [71,72], we surprisingly found that the pro-inflammatory FAs (PA and AA) expanded Bregs in ULNs. Collectively, we propose a hypothesis that immune cells in ULNs may exist in a ‘lipid-starved’ state. Under this paradigm, supplemented AA and PA are redirected toward energy metabolism such as FA β-oxidation rather than inflammatory mediator synthesis, reshaping immune cell subpopulation homeostasis in ULNs through metabolic reprogramming.

Our study provides the first systematic revelation of FA saturation-dependent modulation of immune responses across multiple maternal immune compartments. While our study administered specific FAs exogenously, it is important to consider their physiological relevance. The FAs we investigated (PA, OA, AA) are not exogenous compounds but are among the most abundant endogenous lipids present at the maternal–fetal interface [73]. Their circulating and tissue levels fluctuate dynamically during normal pregnancy and are often dysregulated in miscarriage. Therefore, our supplementation model does not introduce novel entities but rather modulates the levels of native FAs to mimic a state of nutritional imbalance or stress. This approach allows us to dissect the specific immunomodulatory capacity of each FA class within the complex gestational immune landscape. Despite tissue-specific divergences, the three major FAs collectively exhibited conserved immunomodulatory properties with cross-tissue conservation, as evidenced by OA’s capacity to drive a pan-tissue anti-inflammatory phenotype of multiple immune cell lineages, whereas PA and AA collectively promoted pro-inflammatory response. Previous studies have also shown the similar properties of PA, OA, and AA. For example, PA induces macrophage mitochondrial dysfunction, promotes their M1 polarization, and ultimately triggers adipose tissue insulin resistance in gestational diabetes [18]. OA inhibits the pro-inflammatory processes in macrophages while increasing the release of AA [19]. AA hampers the surface expression of cytotoxicity receptors on NK cells, reducing the intracellular signaling proteins activated by these receptors to promote ovarian cancer [20]. To address the possibility that the observed fetal resorption constituted a direct toxic effect rather than immunologically programmed pregnancy failure, we critically evaluated our experimental timeline and administration protocol. The treatment window (GD0.5–7.5) preceded the typical onset of significant embryo resorption in mice (after GD9.5) [74], supporting the interpretation of early immunomodulatory programming rather than acute lipotoxicity. Furthermore, the intraperitoneally administered FAs were conjugated with BSA to mimic physiological transport, and the doses used were substantially lower than physiological plasma concentrations [21], thereby minimizing the risk of nonspecific stress or toxicity. Collectively, these considerations strengthen the conclusion that PA, OA, and AA differentially modulate pregnancy outcomes via structured immune reprogramming rather than direct embryonic damage. Overall, our research confirms that FA types constitute pivotal determinants driving interorganizational immune phenotypic heterogeneity and influencing pregnancy outcomes, with bidirectional modulatory effects on both maternal–fetal interface and offspring immune homeostasis. Comprehensive evaluation of FA proportion and subtypes during gestational nutritional programming requires urgent investigation to equilibrate the maternal–fetal immune cross-talk networks.

However, our study presents limitations that require further address. Currently, we focus exclusively on the phenotypic alterations modulated by FA subtypes across immune cell lineages without elucidating the underlying molecular mechanism. Also, we chose intraperitoneal injection to supply FAs in mice, an approach that may not completely simulate the natural metabolic process of nutrients. And the MR analysis is limited by the scarcity of GWAS data available for PA, OA, and AA [75,76]. This dearth of genetic data may have contributed to our inability to establish causal relationships. Future studies, potentially with more extensive genetic data or clinical trials, would be necessary to further elucidate these associations.

## Conclusions

In summary, our findings indicate distinct roles of FA subtypes (PA/OA/AA) in pregnancy outcomes, with AA promoting embryo resorption and reducing fetal and placental weights, while PA and OA making no difference. Further examination of maternal–fetal interface and peripheral immune networks reveals PA predominantly activates splenic NK and monocyte responses without disrupting core maternal–fetal tolerance. OA consistently promotes immunosuppression by expanding anti-inflammatory macrophages, regulatory NK cells, and Th2 responses across multiple tissue compartments. In striking contrast, AA drives potent decidual inflammation through cytotoxic NK and T cell activation, yet paradoxically induces tolerogenic adaptations in ULNs, revealing its unique context-dependent immunomodulation. Collectively, our findings establish immune–metabolic checkpoints as novel targets for lipid-based nutritional regimens during gestation, while delineating precision lipid supplementation protocols – particularly ω-9 MUFA enrichment coupled with ω-6 PUFA restriction – as a translatable strategy to mitigate inflammatory priming and thereby prevent immune-mediated gestational complications.

**Clinical Perspectives**

- Fatty acids act as both metabolic fuels and immune signaling molecules during pregnancy, yet how diverse fatty acids program maternal immunity and influence pregnancy outcomes remains unclear.
- Arachidonic acid increased the embryo resorption rates and decreased the fetal and placental weights, consistent with its role in systemic pro-inflammatory priming. In contrast, oleic acid enforced broad immunosuppression, while palmitic acid activated immunity primarily in the spleen.
- Tissue-specific immune reprogramming by fatty acids may be a mechanistic basis for miscarriage, thus dietary modulation could be a targeted clinical strategy to prevent recurrent pregnancy loss.

## Supplementary material

online supplementary material 1.

online supplementary material 2.

online supplementary material 3.

online supplementary material 4.

online supplementary material 5.

online supplementary material 6.

## Data Availability

All of the information collected or analyzed during the present study is included in this published article and its supplementary files.

## Open Access

This article has been published open access under our Subscribe to Open programme, made possible through the support of our subscribing institutions, learn more here: https://portlandpress.com/pages/open_access_options_and_prices#conditional

## Abbreviations

AA: arachidonic acid
APC: antigen-presenting cells
BSA: bovine serum albumin
Breg: regulatory B cell
CTL: cytotoxic T lymphocyte
DC: dendritic cell
DIC: decidual immune cell
FA: fatty acid
FCM: flow cytometry
GDM: gestational diabetes
MR: Mendelian randomization
MUFA: monosaturated fatty acid
NK: natural killer cells
OA: oleic acid
PA: palmitic acid
PUFA: polyunsaturated fatty acid
RSA: recurrent spontaneous abortion
SPF: specific pathogen free
SSC: side scatter
ULN: uterine-draining lymph node
cDC: conventional dendritic cell
dMφ: decidual macrophage
pDC: plasmacytoid dendritic cell
