# Supplementary material for: Differential modulation of gestational immunity by fatty acids: tissue-specific immune remodeling and clinical implications
Source: Clin Sci (Lond). 2026 Jan 9;140(1):47–64. doi: 10.1042/CS20257900 (PMC12862962; doi:10.1042/CS20257900)
Supplement: online supplementary material 1. [file cs-140-1-CS20257900-s001.docx]

**Supplementary file 1. Details of studies and datasets used for MR analysis.**

| **Exposure/Outcome** | **Consortium or cohort study** | **Participants** | **PMID/Web Source** |
| --- | --- | --- | --- |
| Arachidonic acid | CHARGE consortium | 8,631 individuals of European ancestry | PMID: 24823311 |
| Oleic acid | CHARGE consortium | 8,961 individuals of European ancestry | PMID: 23362303 |
| Palmitic acid | CHARGE consortium | 8,961 individuals of European ancestry | PMID: [23362303](https://pubmed.ncbi.nlm.nih.gov/23362303) |
| Number of spontaneous miscarriages | UK Biobank | 78,700 individuals of European ancestry | [Trait: Number of spontaneous miscarriages - IEU OpenGWAS project (mrcieu.ac.uk)](https://gwas.mrcieu.ac.uk/datasets/ukb-b-419/) |
| Recurrent spontaneous miscarriage | FinnGen | 112234 individuals of European ancestry | https://r10.risteys.finngen.fi/endpoints/N14_HABITABORT |

CHARGE, Cohorts for Heart and Aging Research in Genomic Epidemiology; GWAS, genome-wide association study; Finngen, Finnish Genome Center; UK Biobank: United Kingdom Biobank
