# Supplementary material for: Differential modulation of gestational immunity by fatty acids: tissue-specific immune remodeling and clinical implications
Source: Clin Sci (Lond). 2026 Jan 9;140(1):47–64. doi: 10.1042/CS20257900 (PMC12862962; doi:10.1042/CS20257900)
Supplement: online supplementary material 2. [file cs-140-1-CS20257900-s002.docx]

**Supplementary file 2. Detailed information on genetic instruments and associations with fatty acid and the studied outcomes.**

| Exposure | SNP | Chr | EA | NEA | EAF | Fatty acids | | | | | Number of spontaneous miscarriages | | | Recurrent spontaneous miscarriage | | |
| --- | --- | --- | --- | --- | --- | --- | --- | --- | --- | --- | --- | --- | --- | --- | --- | --- |
|  |  |  |  |  |  | Explained variance (R2) | F statistic | Beta | SE | P | Beta | SE | P | Beta | SE | P |
| Arachidonic acid | rs12209128 | 6 | T | G | 0.411 | 0.010894 | 95.03576 | -0.15 | 0.031935 | 2.64E-06 | 0.005775 | 0.003683 | 0.12 | 0.021791 | 0.057908 | 2.64E-06 |
|  | rs12471016 | 2 | T | C | 0.1002 | 0.01127 | 98.35727 | -0.25 | 0.054114 | 3.84E-06 | 0.010264 | 0.006307 | 0.1 | -0.03692 | 0.069996 | 3.84E-06 |
|  | rs16829840 | 3 | T | C | 0.02249 | 0.009304 | 81.03568 | 0.46 | 0.082568 | 2.53E-08 | 0.006983 | 0.012001 | 0.56 | -0.05607 | 0.227048 | 2.53E-08 |
|  | rs1741 | 16 | C | G | 0.2986 | 0.016755 | 147.043 | -0.2 | 0.03129 | 1.64E-10 | -0.00361 | 0.003915 | 0.36 | 0.08757 | 0.060526 | 1.64E-10 |
|  | rs17663676 | 11 | T | C | 0.97137 | 0.012287 | 107.3399 | -0.47 | 0.085935 | 4.52E-08 | 0.009927 | 0.009175 | 0.28 | 0.114437 | 0.143803 | 4.52E-08 |
|  | rs2269928 | 11 | T | G | 0.7853 | 0.679946 | 18332.06 | 1.42 | 0.045781 | 1.00E-200 | 0.002392 | 0.004365 | 0.58 | 0.097718 | 0.084218 | 1.00E-200 |
|  | rs274557 | 5 | T | C | 0.5859 | 0.009511 | 82.85629 | 0.14 | 0.030182 | 3.51E-06 | 0.004845 | 0.003691 | 0.19 | 0.015104 | 0.056129 | 3.51E-06 |
|  | rs9394931 | 6 | T | C | 0.408 | 0.010869 | 94.82025 | -0.15 | 0.031745 | 2.30E-06 | 0.002265 | 0.003645 | 0.53 | 0.12555 | 0.056983 | 2.30E-06 |
| Oleic acid | rs102275 | 11 | T | C | 0.6309 | 0.024637 | 226.2995 | -0.23 | 0.019412 | 2.19E-32 | 0.007523 | 0.003748 | 0.045 | 0.025222 | 0.056845 | 0.65726 |
|  | rs11006464 | 10 | T | C | 0.9816 | 0.005304 | 47.77522 | 0.3832 | 0.083822 | 4.84E-06 | -0.01033 | 0.01157 | 0.37 | -0.21353 | 0.423079 | 0.613775 |
|  | rs12529874 | 6 | A | G | 0.03579 | 0.015895 | 144.7048 | -0.4799 | 0.093204 | 2.62E-07 | 0.010636 | 0.010128 | 0.29 | 0.05587 | 0.168626 | 0.740398 |
|  | rs17648246 | 13 | A | G | 0.97342 | 0.003913 | 35.1976 | -0.275 | 0.056476 | 1.12E-06 | 0.013012 | 0.009821 | 0.19 | -0.01711 | 0.138426 | 0.901618 |
|  | rs17774576 | 10 | C | G | 0.8865 | 0.030467 | 281.5295 | 0.3891 | 0.085104 | 4.83E-06 | 0.006554 | 0.005783 | 0.26 | -0.09946 | 0.085161 | 0.242839 |
|  | rs3134950 | 6 | A | C | 0.6268 | 0.004178 | 37.58738 | -0.0945 | 0.019421 | 1.14E-06 |  |  |  | -0.07384 | 0.056747 | 0.193185 |
|  | rs334809 | 3 | A | T | 0.3231 | 0.047231 | 444.1181 | 0.3286 | 0.067917 | 1.31E-06 | -0.00181 | 0.004088 | 0.66 | -0.02595 | 0.060431 | 0.667566 |
|  | rs4731889 | 7 | A | G | 0.4315 | 0.003595 | 32.32305 | -0.0856 | 0.018679 | 4.59E-06 | -0.00193 | 0.003591 | 0.59 | 0.04772 | 0.056861 | 0.401335 |
| Palmitic acid | rs10234749 | T | T | G | 0.2413 | 0.011706 | 106.1123 | -0.1788 | 0.038863 | 4.21E-06 | -0.00491 | 0.004268 | 0.25 | 0.023677 | 0.05928 | 0.689595 |
|  | rs10414689 | T | T | C | 0.98875 | 0.013598 | 123.4997 | 0.7818 | 0.159172 | 9.03E-07 | 0.009336 | 0.017806 | 0.6 | -0.3227 | 0.828951 | 0.697066 |
|  | rs10809457 | T | T | G | 0.4335 | 0.009544 | 86.33145 | -0.1394 | 0.028415 | 9.30E-07 | -0.00262 | 0.003635 | 0.47 | 0.014257 | 0.056876 | 0.802068 |
|  | rs12297524 | T | T | C | 0.3221 | 0.006911 | 62.3475 | 0.1258 | 0.027512 | 4.82E-06 | -0.00094 | 0.003691 | 0.8 | 0.009859 | 0.05988 | 0.869221 |
|  | rs1980946 | C | C | G | 0.90798 | 0.01484 | 134.9502 | -0.298 | 0.064929 | 4.44E-06 | -0.00722 | 0.006654 | 0.28 | 0.043037 | 0.100798 | 0.66941 |
|  | rs2391388 | A | A | C | 0.5368 | 0.015668 | 142.602 | -0.1775 | 0.026648 | 2.72E-11 | -0.00171 | 0.003591 | 0.630001 | 0.111232 | 0.058065 | 0.055412 |
|  | rs603424 | A | A | G | 0.1953 | 0.01124 | 101.8399 | 0.1891 | 0.036115 | 1.64E-07 | -0.00013 | 0.0047 | 0.98 | -0.12458 | 0.087807 | 0.155969 |
|  | rs7561966 | A | A | G | 0.8262 | 0.133147 | 1376.087 | -0.6809 | 0.146173 | 3.19E-06 | 0.003624 | 0.004657 | 0.44 | 0.062762 | 0.067614 | 0.353288 |
|  | rs780093 | T | T | C | 0.4162 | 0.007727 | 69.76777 | 0.1261 | 0.026851 | 2.65E-06 | 0.001302 | 0.003668 | 0.719999 | -0.00619 | 0.058964 | 0.916435 |
|  | rs9816269 | T | T | C | 0.95808 | 0.010801 | 97.82522 | 0.3667 | 0.080058 | 4.64E-06 | 0.001136 | 0.009989 | 0.91 | 0.007799 | 0.140089 | 0.955602 |
|  | rs994988 | T | T | C | 0.5153 | 0.009721 | 87.94548 | -0.1395 | 0.026877 | 2.10E-07 | -0.00209 | 0.003575 | 0.56 | -0.02986 | 0.056259 | 0.595548 |

EA, effect allele; EAF, effect allele frequency; NEA, non-effect allele; SE, standard error; SNP, single nucleotide polymorphism; Chr, chromosome
