# Supplementary material for: Differential modulation of gestational immunity by fatty acids: tissue-specific immune remodeling and clinical implications
Source: Clin Sci (Lond). 2026 Jan 9;140(1):47–64. doi: 10.1042/CS20257900 (PMC12862962; doi:10.1042/CS20257900)
Supplement: online supplementary material 3. [file cs-140-1-CS20257900-s003.docx]

**Supplementary file 3. Results of SNPs related to known phenotypes.**

| **Exposure** | **SNP** | **Trait** |
| --- | --- | --- |
| Arachidonic acid | rs12209128 | Omega-6 polyunsaturated fatty acid measurement |
|  | rs12471016 | Omega-6 polyunsaturated fatty acid measurement |
|  | rs16829840 | Omega-6 polyunsaturated fatty acid measurement |
|  | rs1741 | Body height  Phosphatidylcholine measurement  Cholesteryl ester 20:3 measurement  Phosphatidylethanolamine measurement  1-oleoyl-2-dihomo-linolenoyl-gpc (18:1/20:3) measurement  1-stearoyl-2-dihomo-linolenoyl-gpc (18:0/20:3n3 or 6) measurement  1-dihomo-linolenoyl-gpc (20:3n3 or 6) measurement  1-palmitoyl-2-dihomo-linolenoyl-gpc (16:0/20:3n3 or 6) measurement  Omega-6 polyunsaturated fatty acid measurement |
|  | rs17663676 | / |
|  | rs2269928 | Omega-3 polyunsaturated fatty acid measurement  Omega-6:omega-3 polyunsaturated fatty acid ratio  Low density lipoprotein cholesterol measurement |
|  | rs274557 | Omega-6 polyunsaturated fatty acid measurement |
|  | rs9394931 | Omega-6 polyunsaturated fatty acid measurement |
| Oleic acid | rs102275 | Coronary artery calcification  Serum metabolite measurement  Irritability measurement  Phosphatidylcholine measurement  Erythrocyte count  Phospholipid measurement, high density lipoprotein cholesterol measurement  High density lipoprotein particle size measurement  Omega-3 polyunsaturated fatty acid measurement  High density lipoprotein cholesterol measurement  Esterified cholesterol measurement, high density lipoprotein cholesterol measurement  Free cholesterol measurement, high density lipoprotein cholesterol measurement  Lipid measurement, high density lipoprotein cholesterol measurement  Unipolar depression, bipolar disorder  Fatty acid measurement  Eosinophil count  Reticulocyte count  Red blood cell density measurement  Omega-6 polyunsaturated fatty acid measurement  Phosphatidylcholine 36:4 measurement  Palmitoleic acid measurement  Oleic acid measurement  Crohn's disease  High density lipoprotein cholesterol measurement, metabolic syndrome  Phospholipid measurement  Alpha-linolenic acid measurement  Docosapentaenoic acid measurement  Cis/trans-18:2 fatty acid measurement, trans fatty acid measurement  Unipolar depression  Qt interval  Lysophosphatidylcholine measurement  Diglyceride measurement  Triacylglycerol 56:6 measurement  Triacylglycerol 56:8 measurement  Cholesteryl ester 22:5 measurement  Cholesteryl ester 20:3 measurement  Cholesteryl ester 20:5 measurement  Phosphatidylethanolamine measurement  Diacylglycerol 36:2 measurement  Diacylglycerol 36:3 measurement  Cholesteryl ester 20:4 measurement  Lysophosphatidylethanolamine measurement  Phosphatidate measurement  Phosphatidylinositol measurement  Triglyceride measurement  Sphingomyelin measurement  Glycerophospholipid measurement  Cholesteryl ester 18:3 measurement  Cholesteryl ester 16:0 measurement  Cholesteryl ester 18:2 measurement  Phosphatidylserines measurement  Cholesteryl ester 14:0 measurement  Cholesteryl ester 22:6 measurement  Cholesteryl ester measurement  Diacylglycerol 38:3 measurement  Diacylglycerol 38:4 measurement  Diacylglycerol 38:5 measurement  Choline measurement  Cholesteryl ester measurement, intermediate density lipoprotein measurement  Low density lipoprotein cholesterol measurement, lipid measurement  Low density lipoprotein cholesterol measurement, phospholipid measurement  Total cholesterol measurement, low density lipoprotein cholesterol measurement  Cholesterol:total lipids ratio, low density lipoprotein cholesterol measurement  Free cholesterol measurement, intermediate density lipoprotein measurement  Low density lipoprotein cholesterol measurement, phospholipids:total lipids ratio  Low density lipoprotein cholesterol measurement  Total cholesterol measurement, intermediate density lipoprotein measurement  Intermediate density lipoprotein measurement  Total cholesterol measurement, very low density lipoprotein cholesterol measurement  Cholesteryl ester measurement, low density lipoprotein cholesterol measurement  Free cholesterol measurement, low density lipoprotein cholesterol measurement  Cholesterol:total lipids ratio, intermediate density lipoprotein measurement  Cholesteryl esters:total lipids ratio, intermediate density lipoprotein measurement  Free cholesterol measurement, very low density lipoprotein cholesterol measurement  Total cholesterol measurement  Triglyceride:hdl cholesterol ratio  Lipid measurement, lipoprotein measurement  Very low density lipoprotein cholesterol measurement, cholesteryl esters:total lipids ratio  Cholesteryl ester measurement, very low density lipoprotein cholesterol measurement  Remnant cholesterol measurement  Lipoprotein measurement, phospholipid measurement  Very low density lipoprotein cholesterol measurement, cholesterol:total lipids ratio  Apolipoprotein b measurement  Polyunsaturated fatty acid measurement  Free cholesterol measurement  Esterified cholesterol measurement  Lysophosphatidylcholine 22:1 measurement  Lysophosphatidylethanolamine 20:4 measurement  Oleoyl-arachidonoyl-glycerol (18:1/20:4) [1] measurement  Oleoyl-arachidonoyl-glycerol (18:1/20:4) [2] measurement  Linoleoyl-arachidonoyl-glycerol (18:2/20:4) [1] measurement  Linoleoyl-arachidonoyl-glycerol (18:2/20:4) [2] measurement  Metabolite measurement  Hydroxypalmitoyl sphingomyelin (d18:1/16:0(oh)) measurement  Arachidonoylcarnitine (c20:4) measurement  1-palmitoleoyl-2-linolenoyl-gpc (16:1/18:3) measurement  1-stearoyl-2-arachidonoyl-gpi (18:0/20:4) measurement  Level of phosphatidylcholine (18:0_20:5) in blood serum  Level of phosphatidylcholine (18:0_22:5) in blood serum  Triacylglycerol 58:7 measurement  Phosphatidylcholine acyl-alkyl c38:4 measurement  Phosphatidylcholine diacyl c40:4 measurement  Phosphatidylcholine diacyl c42:4 measurement  Phosphatidylcholine acyl-alkyl c40:4 measurement  Phosphatidylcholine acyl-alkyl c40:5 measurement  Phosphatidylcholine acyl-alkyl c42:5 measurement  Phosphatidylcholine acyl-alkyl c44:5 measurement  Phosphatidylcholine acyl-alkyl c44:6 measurement  1-arachidonoyl-gpc (20:4n6) measurement  2-arachidonoyl-gpc (20:4) measurement  1-(1-enyl-palmitoyl)-2-arachidonoyl-gpc (p-16:0/20:4) measurement  1-palmitoyl-2-arachidonoyl-gpc (16:0/20:4n6) measurement  Stearoyl-arachidonoyl-glycerol (18:0/20:4) [1] measurement  1-myristoyl-2-arachidonoyl-gpc (14:0/20:4) measurement  1-stearoyl-2-arachidonoyl-gpc (18:0/20:4) measurement  1-(1-enyl-stearoyl)-2-arachidonoyl-gpe (p-18:0/20:4) measurement  Total phospholipids in lipoprotein particles measurement  Phospholipids in ldl measurement  Phospholipids in hdl measurement  1,2-dipalmitoyl-gpc (16:0/16:0) measurement  Cholesterol in medium hdl measurement  Free cholesterol in medium hdl measurement  1-(1-enyl-palmitoyl)-2-oleoyl-gpc (p-16:0/18:1) measurement  Sphingomyelin (d18:1/22:1, d18:2/22:0, d16:1/24:1) measurement  Phospholipids:total lipids ratio  1-(1-enyl-palmitoyl)-2-palmitoleoyl-gpc (p-16:0/16:1) measurement  1-(1-enyl-palmitoyl)-2-palmitoyl-gpc (p-16:0/16:0) measurement  Total lipids in lipoprotein particles measurement  Free cholesterol in ldl measurement  Free cholesterol in hdl measurement  Phosphoglycerides measurement  Saturated fatty acids measurement  Cholesterol in large ldl measurement  Free cholesterol in large ldl measurement  Free cholesterol in medium ldl measurement  Phospholipids in small ldl measurement  Arachidonate 20:4n6 measurement  Total lipids in idl  Cholesterol in idl measurement  Cholesterol in medium ldl measurement  Total lipids in ldl measurement  Total lipids in hdl measurement  Lipoprotein measurement  Apolipoprotein a 1 measurement  Cholesterol in medium vldl measurement  Phospholipids in large ldl measurement  Free cholesterol in idl measurement  Total lipids in large ldl  Free cholesterol in small ldl measurement  Cholesterol to total lipids in idl percentage  Cholesteryl esters to total lipids in idl percentage  Triglycerides to total lipids in medium ldl percentage  Cholesterol to total lipids in very small vldl percentage  Free cholesterol to total lipids in very small vldl percentage  Cholesterol to total lipids in medium ldl percentage  Peptic ulcer disease, bipolar disorder  Level of phosphatidylcholine (o-16:0_20:4) in blood serum  Level of phosphatidylcholine (17:0_20:4) in blood serum  Level of phosphatidylcholine (16:0_20:5) in blood serum  Level of phosphatidylcholine (16:0_22:5) in blood serum  Level of phosphatidylcholine (16:1_20:4) in blood serum  Level of phosphatidylcholine (18:0_20:4) in blood serum  Level of phosphatidylcholine (o-18:2_18:1) in blood serum  Level of phosphatidylcholine (16:0_16:0) in blood serum  Level of sterol ester (27:1/16:0) in blood serum  Level of phosphatidylcholine (20:4_0:0) in blood serum  Protein measurement  Carboxypeptidase b measurement  Oleoyl-oleoyl-glycerol (18:1/18:1) [1] measurement  1-palmitoyl-2-linoleoyl-gpi (16:0/18:2) measurement |
|  | rs11006464 | Fatty acid measurement |
|  | rs12529874 | Fatty acid measurement |
|  | rs17648246 | Fatty acid measurement |
|  | rs17774576 | Fatty acid measurement |
|  | rs3134950 | Risk-taking behaviour  Omega-6 polyunsaturated fatty acid measurement  Fatty acid measurement  Phosphatidylcholine measurement  Level of phosphatidylcholine (16:0_16:0) in blood serum |
|  | rs334809 | Fatty acid measurement |
|  | rs4731889 | Fatty acid measurement |
| Palmitic acid | rs10234749 | Fatty acid measurement |
|  | rs10414689 | Fatty acid measurement |
|  | rs10809457 | Fatty acid measurement |
|  | rs12297524 | Fatty acid measurement |
|  | rs1980946 | Fatty acid measurement |
|  | rs2391388 | Fatty acid measurement  Phosphatidylcholine measurement  Lysophosphatidylcholine measurement |
|  | rs603424 | Heel bone mineral density  Coronary artery disease  Fatty acid desaturase enzyme activity measurement  Serum metabolite measurement  Low density lipoprotein cholesterol measurement  Obsolete_red blood cell distribution width  Cardiovascular disease  Lymphocyte count  Monocyte count  Mean platelet volume  Systolic blood pressure  Alkaline phosphatase measurement  Palmitoleic acid measurement  Vaccenic acid measurement  Diastolic blood pressure  Fatty acid measurement  Metabolite measurement  Phospholipid measurement  Bmi-adjusted waist circumference  Apolipoprotein b measurement  Total cholesterol measurement  Hba1c measurement  Phosphatidylcholine measurement  Carnitine measurement  Lysophosphatidylcholine measurement  Ceramide measurement  Diglyceride measurement  Triacylglycerol 48:3 measurement  Triacylglycerol 50:1 measurement  Acylcarnitine measurement  Lysophosphatidylcholine 16:1 measurement  Cholesteryl ester 16:1 measurement  Low density lipoprotein particle size measurement  Low density lipoprotein cholesterol measurement, phospholipids:total lipids ratio  Triacylglycerol 50:2 measurement  Triacylglycerol 50:3 measurement  Triacylglycerol 51:1 measurement  Triacylglycerol 52:3 measurement  Lysophosphatidylcholine 17:1 measurement  Hemoglobin a1 measurement  1-palmitoleoyl-gpc (16:1) measurement  Stearic acid measurement  Level of phosphatidylcholine (o-18:0_16:1) in blood serum  Level of phosphatidylcholine (16:1_18:1) in blood serum  Level of phosphatidylcholine (16:1_18:2) in blood serum  Level of sterol ester (27:1/16:1) in blood serum  Myristate 14:0-to-myristoleate 14:1n5 ratio  Margarate 17:0 measurement  Stearate 18:0 measurement  Myristoleate 14:1n5 measurement  Palmitoleate 16:1n7 measurement  5-dodecenoate 12:1n7 measurement  1-(1-enyl-palmitoyl)-2-palmitoleoyl-gpc (p-16:0/16:1) measurement  5-dodecenoylcarnitine (c12:1) measurement  9-hydroxystearate measurement  Palmitoleoylcarnitine (c16:1) measurement  Palmitoleoyl-linoleoyl-glycerol (16:1/18:2) [1] measurement  1-(1-enyl-stearoyl)-2-arachidonoyl-gpc (p-18:0/20:4) measurement  Decadienedioic acid (c10:2-dc) measurement  Bone density  1-palmitoleoylglycerol (16:1) measurement  1-stearyl-gpc (o-18:0) measurement  1-(1-enyl-stearoyl)-2-oleoyl-gpc (p-18:0/18:1) measurement  Stearoylcarnitine measurement  2-palmitoleoyl-gpc (16:1) measurement  Tetradecadienedioate (c14:2-dc) measurement  Monounsaturated fatty acids; 16:1, 18:1 measurement  Saturated fatty acids to total fatty acids percentage  Ecosanoids measurement  Body height |
|  | rs7561966 | Fatty acid measurement |
|  | rs780093 | Low density lipoprotein cholesterol measurement  Total cholesterol measurement  Triglyceride measurement  Creatinine measurement, glomerular filtration rate  Glomerular filtration rate  Urinary albumin to creatinine ratio  Hypertriglyceridemia  Serum metabolite measurement  Nephrolithiasis  Leucine measurement  Isoleucine measurement  Very low density lipoprotein particle size measurement  Very low density lipoprotein cholesterol measurement  Phospholipid measurement, high density lipoprotein cholesterol measurement  Very low density lipoprotein cholesterol measurement, phospholipid measurement  Triglyceride measurement, very low density lipoprotein cholesterol measurement  Free cholesterol measurement, very low density lipoprotein cholesterol measurement  Very low density lipoprotein cholesterol measurement, lipid measurement  Cardiovascular disease  Reticulocyte measurement  Myeloid white cell count  Sex hormone-binding globulin measurement  Glucose homeostasis measurement, glucose effectiveness measurement  Palmitoleic acid measurement  Drugs used in diabetes use measurement  Triglyceride measurement, metabolic syndrome  Triglyceride measurement, blood pressure  Urate measurement  Crohn's disease  Breast size  Fatty acid measurement  Calcium measurement  Bmi-adjusted leptin measurement  Leptin measurement  Type 2 diabetes mellitus  Glucose measurement  Phosphatidylcholine measurement  Sphingomyelin measurement  Diacylglycerol 34:3 measurement  Diacylglycerol 32.2 measurement  Lysophosphatidylethanolamine measurement  Diacylglycerol 44:7 measurement  Gestational diabetes  Ceramide measurement  Histidine measurement  Retinol measurement  Insulin resistance  Total lipids in hdl measurement  Triacylglycerol 46:2 measurement |
|  | rs9816269 | Fatty acid measurement |
|  | rs994988 | Fatty acid measurement |

SNP, single nucleotide polymorphism
