# Supplementary material for: Differential modulation of gestational immunity by fatty acids: tissue-specific immune remodeling and clinical implications
Source: Clin Sci (Lond). 2026 Jan 9;140(1):47–64. doi: 10.1042/CS20257900 (PMC12862962; doi:10.1042/CS20257900)
Supplement: online supplementary material 4. [file cs-140-1-CS20257900-s004.docx]

**Supplementary file 4. Details of horizontal pleiotropy and heterogeneity.**

| **Exposure** | **Outcome** | **Horizontal Pleiotropy** | | **Heterogeneity** | |
| --- | --- | --- | --- | --- | --- |
|  |  | **P-value (MR-Egger)** | **P-value (MR-PRESSO)** | **P-value (IVW)** | **P-value (MR-Egger)** |
| Arachidonic acid | Number of spontaneous miscarriages | 0.627459 | 0.434000 | 0.199501 | 0.152195 |
|  | Recurrent spontaneous miscarriage | 0.101021 | 0.389667 | 0.23063 | 0.472990 |
| Oleic acid | Number of spontaneous miscarriages | 0.986108 | 0.279000 | 0.263211 | 0.175355 |
|  | Recurrent spontaneous miscarriage | 0.536207 | 0.880667 | 0.844879 | 0.811639 |
| Palmitic acid | Number of spontaneous miscarriages | 0.247445 | 0.723333 | 0.923272 | 0.966349 |
|  | Recurrent spontaneous miscarriage | 0.792837 | 0.906333 | 0.896110 | 0.847003 |

MR-Egger, Mendelian Randomization Egger's method; IVW, Inverse Variance Weighted; MR-PRESSO, Mendelian Randomization Pleiotropy RESidual Sum and Outlier
