# Supplementary material for: Differential modulation of gestational immunity by fatty acids: tissue-specific immune remodeling and clinical implications
Source: Clin Sci (Lond). 2026 Jan 9;140(1):47–64. doi: 10.1042/CS20257900 (PMC12862962; doi:10.1042/CS20257900)
Supplement: online supplementary material 5. [file cs-140-1-CS20257900-s005.docx]

**Supplementary file 5. Summary of phenotypic markers, their associated cell types, and functional relevance.**

| **Phenotypic markers** | **Associated cell types** | **Function** | **References** |
| --- | --- | --- | --- |
| CD86 | Macrophages(M1) | Marker of classically activated (M1) macrophages | (1, 2) |
|  | Dendritic cells/Monocytes | Indicator of maturation and antigen-presenting activity (CD86^high^ denotes mature APCs) | (3) |
| CD206 | Macrophages(M2) | Marker of alternatively activated (anti-inflammatory) macrophages | (4) |
| CD11c | Dendritic cells | Specific marker for conventional DCs | (5) |
|  | Macrophages, NK cells | Inflammatory marker in macrophages (CD11c^+^)and NK cells(NK1.1^+^B220^+^CD11c^+^) | (6, 7) |
| B220 (CD45R) | NK cells | Expressed in a subset of activated NK cells, equivalent to CD56^bright^NK cell (NK1.1^+^B220^+^CD11c^+^) | (6, 8) |
|  | Plasmacytoid dendritic cells | Identification of pDCs in murine (CD11c^low^ PDCA‑1^+^B220^+^) | (9) |
| NKp46 | NK cells | Activating receptor defining NK cell lineage | (10) |
| IFN-γ | T cells; NK cells | Pro-inflammatory cytokine; mediates immune activation | (11-13) |
| TNF-α | T cells | Pro-inflammatory cytokine; mediates immune activation | (12) |
| IL-4 | CD4^+^ T cells (Th2) | Anti-inflammatory cytokine; promotes Th2 skewing | (11, 12) |
| IL-10 | CD4^+^ T cells (Th2), B cells (Breg) | Anti-inflammatory cytokine; increased regulatory responses | (12) |
| Major histocompatibility complex II (MHC-II) | Dendritic cells/Monocytes | Indicator of maturation and antigen-presenting activity (MHC-II^high^ denotes mature APCs) | (14) |
| PDCA-1 | Plasmacytoid dendritic cells | Identification of pDCs in murine (CD11c^low^ PDCA‑1^+^B220^+^) | (9, 15) |
| Ly-6C | Monocytes | Indicator of monocyte maturation; (Ly-6C^high^ marks immature, inflammatory monocytes while Ly-6C^low^ represents mature monocytes) | (16, 17) |

**References**

1. Cutolo, M., Campitiello, R., Gotelli, E.and Soldano, S. (2022) The Role of M1/M2 Macrophage Polarization in Rheumatoid Arthritis Synovitis. *Front Immunol*. **13**, 867260. 10.3389/fimmu.2022.867260

2. Yan, S., Ding, J., Wang, Z., Zhang, F., Li, J., Zhang, Y. et al. (2023) CTRP6 regulates M1 macrophage polarization via the PPAR-γ/NF-κB pathway and reprogramming glycolysis in recurrent spontaneous abortion. *Int Immunopharmacol*. **124**, 110840. 10.1016/j.intimp.2023.110840

3. Kim, M.K.and Kim, J. (2019) Properties of immature and mature dendritic cells: phenotype, morphology, phagocytosis, and migration. *RSC Adv*. **9**, 11230-11238. 10.1039/c9ra00818g

4. Ono, Y., Yoshino, O., Hiraoka, T., Sato, E., Fukui, Y., Ushijima, A. et al. (2020) CD206+ M2-Like Macrophages Are Essential for Successful Implantation. *Front Immunol*. **11**, 557184. 10.3389/fimmu.2020.557184

5. Taglauer, E.S., Fernandez-Gonzalez, A., Willis, G.R., Reis, M., Yeung, V., Liu, X. et al. (2021) Mesenchymal stromal cell-derived extracellular vesicle therapy prevents preeclamptic physiology through intrauterine immunomodulation†. *Biol Reprod*. **104**, 457-467. 10.1093/biolre/ioaa198

6. Blasius, A.L., Barchet, W., Cella, M.and Colonna, M. (2007) Development and function of murine B220+CD11c+NK1.1+ cells identify them as a subset of NK cells. *J Exp Med*. **204**, 2561-2568. 10.1084/jem.20070991

7. Li, Y., Sang, Y., Chang, Y., Xu, C., Lin, Y., Zhang, Y. et al. (2024) A Galectin-9-Driven CD11c(high) Decidual Macrophage Subset Suppresses Uterine Vascular Remodeling in Preeclampsia. *Circulation*. **149**, 1670-1688. 10.1161/circulationaha.123.064391

8. Mallidi, T.V., Craig, L.E., Schloemann, S.R.and Riley, J.K. (2009) Murine endometrial and decidual NK1.1+ natural killer cells display a B220+CD11c+ cell surface phenotype. *Biol Reprod*. **81**, 310-318. 10.1095/biolreprod.109.076448

9. Tahoori, M.T., Pourfathollah, A.A., Soleimani, M., Vasheghani-Farahani, E., Mohammadzadeh, A., Amari, A. et al. (2015) Fibroblasts feeder niche and Flt3 Ligand as a novel inducer of plasmacytoid dendritic cells development in vitro. *Int Immunopharmacol*. **24**, 474-480. 10.1016/j.intimp.2014.10.031

10. Li, Y., Chen, J., Lin, Y., Xu, L., Sang, Y., Li, D. et al. (2021) Obesity Challenge Drives Distinct Maternal Immune Response Changes in Normal Pregnant and Abortion-Prone Mouse Models. *Front Immunol*. **12**, 694077. 10.3389/fimmu.2021.694077

11. Liu, L., Huang, X., Xu, C., Chen, C., Zhao, W., Li, D. et al. (2020) Decidual CD8(+)T cells exhibit both residency and tolerance signatures modulated by decidual stromal cells. *J Transl Med*. **18**, 221. 10.1186/s12967-020-02371-3

12. Wang, S., Li, M., Sun, F., Chen, C., Ye, J., Li, D. et al. (2021) Altered frequency and function of spleen CTLA-4+Tim-3+ T cells are associated with miscarriage†. *Biol Reprod*. **104**, 410-417. 10.1093/biolre/ioz076

13. Feyaerts, D., Benner, M., van Cranenbroek, B., van der Heijden, O.W.H., Joosten, I.and van der Molen, R.G. (2017) Human uterine lymphocytes acquire a more experienced and tolerogenic phenotype during pregnancy. *Sci Rep*. **7**, 2884. 10.1038/s41598-017-03191-0

14. Blois, S.M., Kammerer, U., Alba Soto, C., Tometten, M.C., Shaikly, V., Barrientos, G. et al. (2007) Dendritic cells: key to fetal tolerance? *Biol Reprod*. **77**, 590-598. 10.1095/biolreprod.107.060632

15. Kadowaki, T., Morishita, A., Niki, T., Hara, J., Sato, M., Tani, J. et al. (2013) Galectin-9 prolongs the survival of septic mice by expanding Tim-3-expressing natural killer T cells and PDCA-1+ CD11c+ macrophages. *Crit Care*. **17**, R284. 10.1186/cc13147

16. Sunderkotter, C., Nikolic, T., Dillon, M.J., Van Rooijen, N., Stehling, M., Drevets, D.A. et al. (2004) Subpopulations of mouse blood monocytes differ in maturation stage and inflammatory response. *J Immunol*. **172**, 4410-4417. 10.4049/jimmunol.172.7.4410

17. Stansfield, B.K.and Ingram, D.A. (2015) Clinical significance of monocyte heterogeneity. *Clin Transl Med*. **4**, 5. 10.1186/s40169-014-0040-3
