# Supplementary material for: Differential modulation of gestational immunity by fatty acids: tissue-specific immune remodeling and clinical implications
Source: Clin Sci (Lond). 2026 Jan 9;140(1):47–64. doi: 10.1042/CS20257900 (PMC12862962; doi:10.1042/CS20257900)
Supplement: online supplementary material 6. [file cs-140-1-CS20257900-s006.pdf]

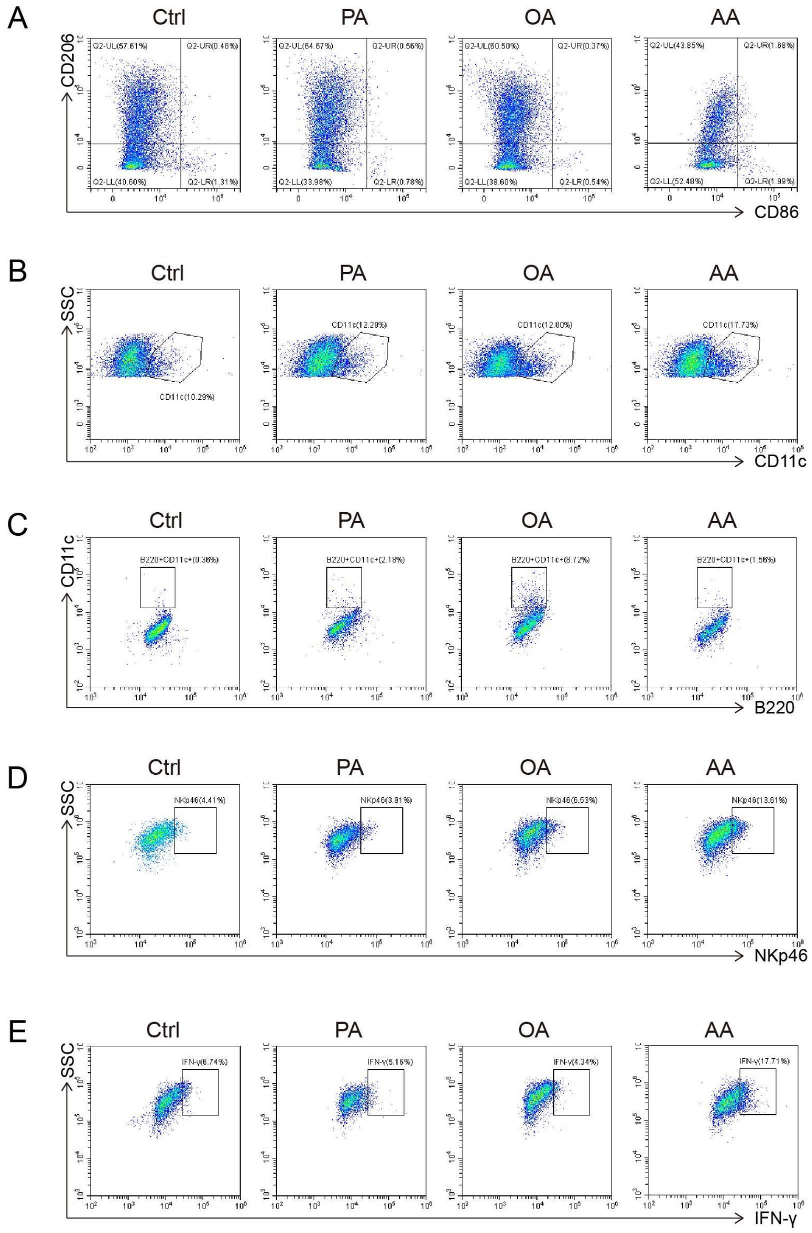

Supplementary Fig.1 FCM gating strategies for decidual innate immune cells.

(A) Representative dot plots of CD86 and CD206 in F4/80^{+} decidual macrophages. (B) Representative dot plots of CD11c in F4/80^{+} decidual macrophages. (C) Representative dot plots of CD11c^{+}B220^{+} counterparts in total CD3^{-}NK1.1^{+} decidual NK cells. (D) Representative dot plots of NKp46 in total CD3^{-}NK1.1^{+} decidual NK cells. (E) Representative dot plots of IFN-γ in total CD3^{-}NK1.1^{+} decidual NK cells.

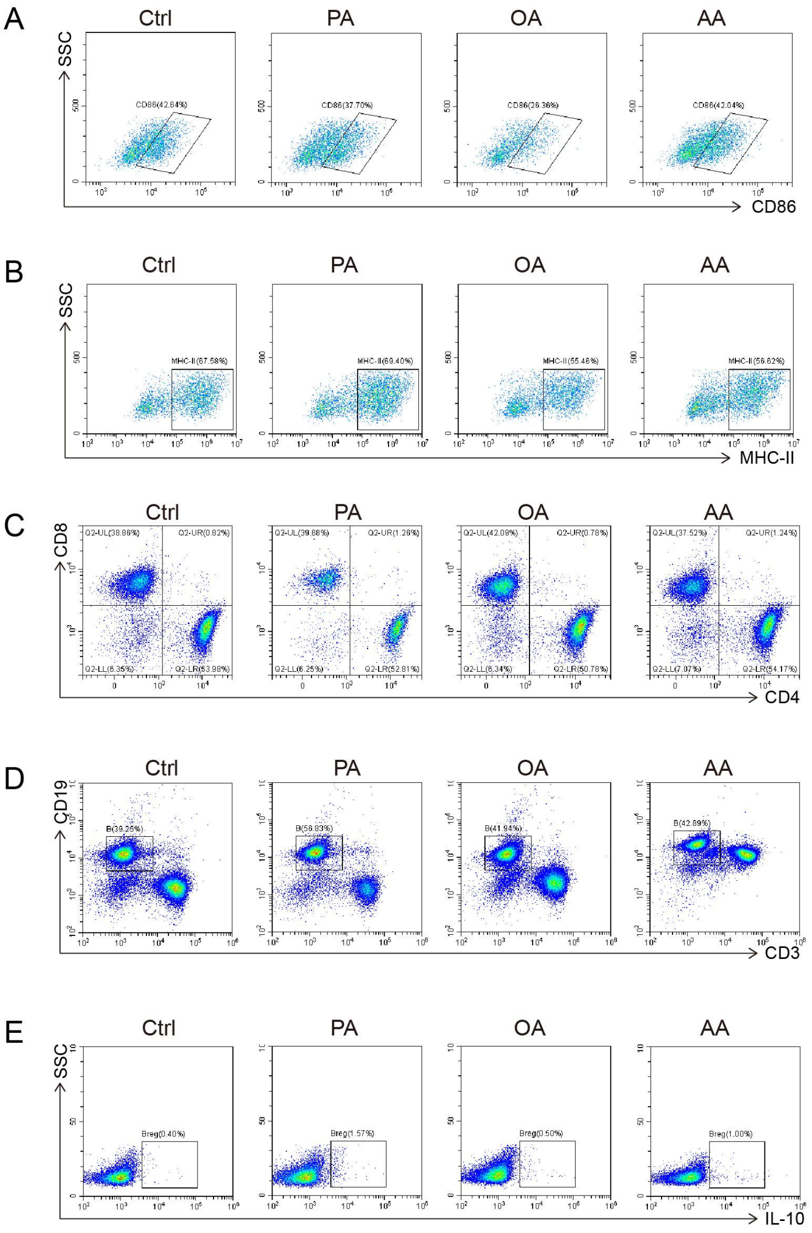

Supplementary Fig.2 FCM gating strategies for ULN immune cells.

(A) Representative dot plots of CD86 in CD11c^{+} DCs. (B) Representative dot plots of MHC-II in CD11c^{+} DCs. (C) Representative dot plots of total CD4^{+}/CD8^{+} T cells. (D) Representative dot plots of total CD3^{-}CD19^{+} B cells. (E) Representative dot plots of IL-10^{+} Breg cells.
